# Supplementary figures and images for: Diversity and ecological structure of vibrios in benthic and pelagic habitats along a latitudinal gradient in the Southwest Atlantic Ocean
Source: PeerJ. 2015 Feb 10;3:e741. doi: 10.7717/peerj.741 (PMC4327252; doi:10.7717/peerj.741)

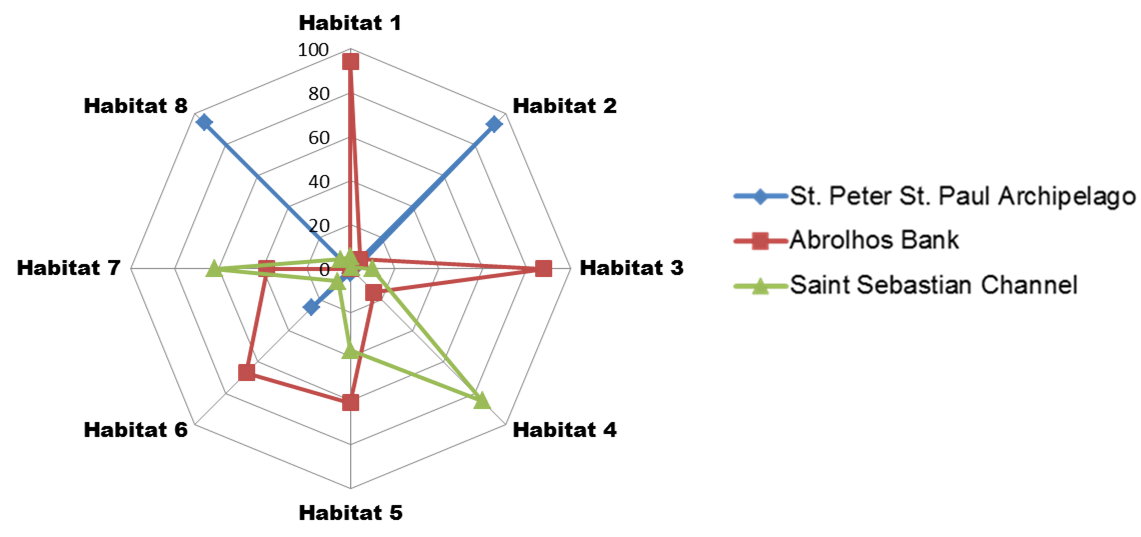

Supplement: Figure S1 — Distribution of the studied regions [Saint Peter and Saint Paul Archipelago (SPSPA), Abrolhos Bank (AB) and Saint Sebastian channel (SS)] in each habitat composition defined by AdaptML approach. Scale represents percentage. [file peerj-03-741-s001.png]

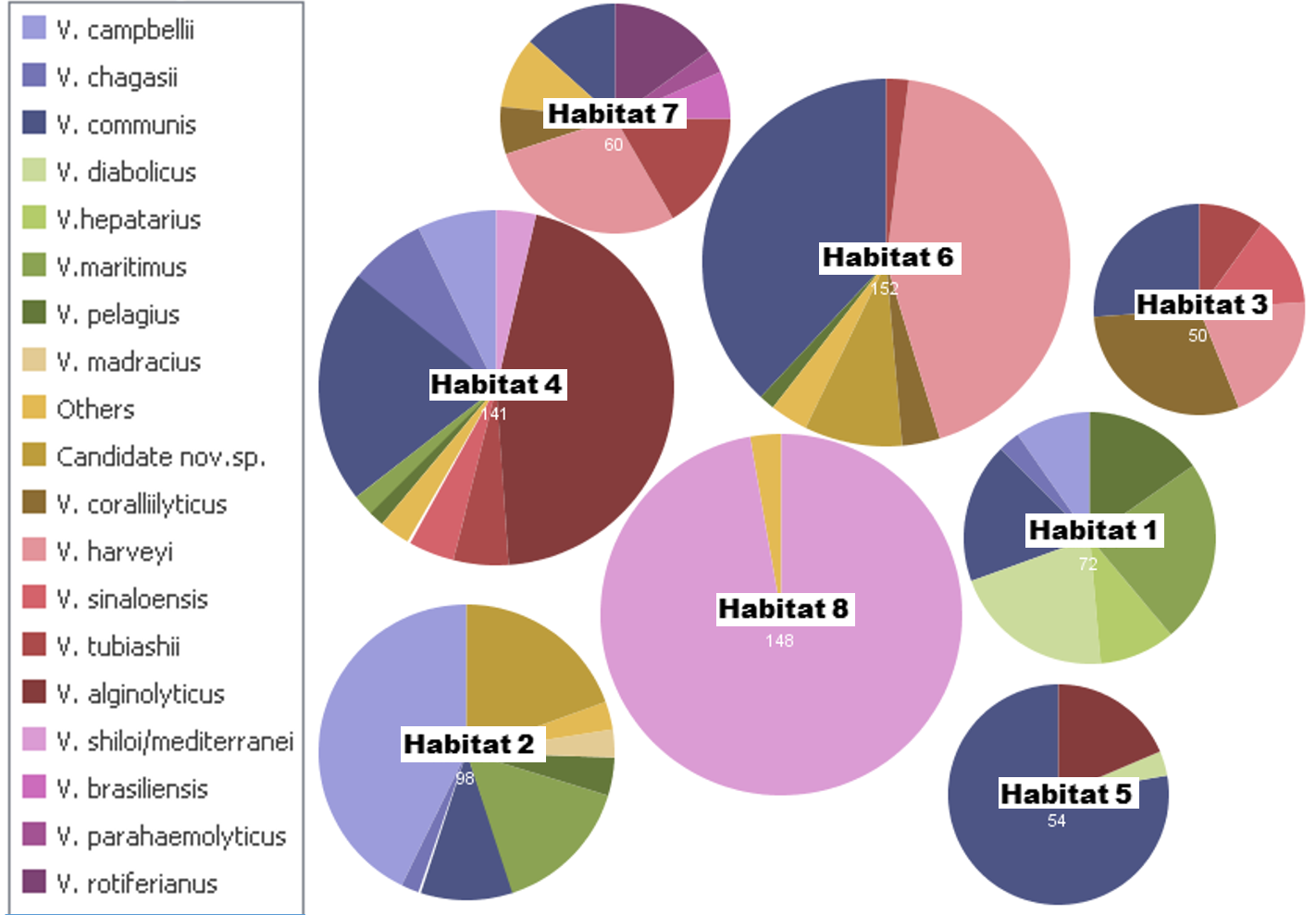

Supplement: Figure S2 — Vibrio species diversity in the habitats. The figure shows how the 775 Vibrio strains are distributed in each habitat. The side of the circle represents the proportion of total strains in each group. Others = represents Vibrio species with low abundance found in that group. Figure generated through Many Eyes website (Viégas et al., 2007). [file peerj-03-741-s002.png]

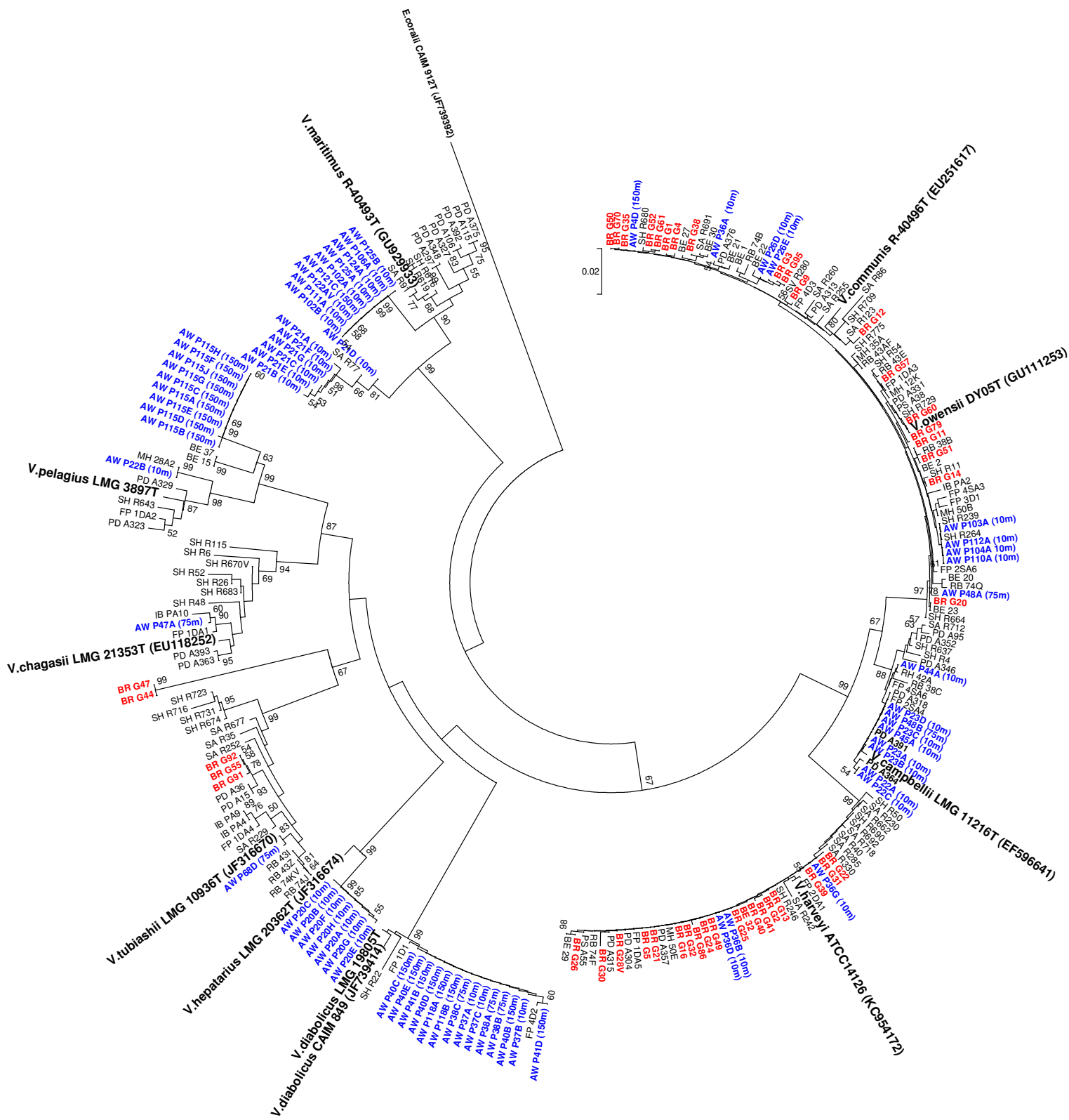

Supplement: Figure S3 — Phylogenetic tree based on the neighbor-joining distance method using pyrH gene sequences showing the relationships among representative Vibrio species from plankton (blue color), rhodoliths (red color) and corals (black). Type strains of Vibrio were included (bold black), Distance estimations were obtained according to the Kimura-2-parameter model. Bootstrap percentages after 1,000 replications are shown. Divergence bar estimated at 2%. Depth is indicated for planktonic strains. [file peerj-03-741-s003.pdf]

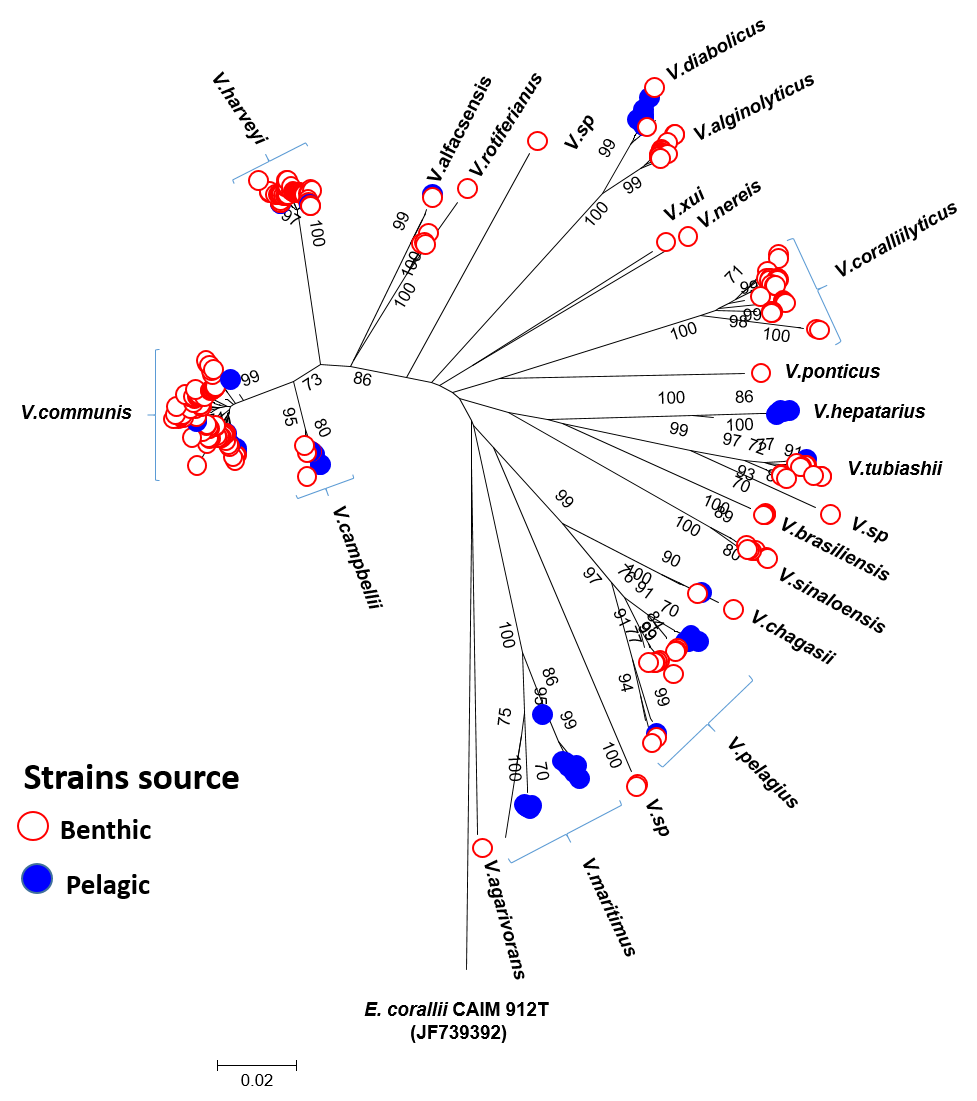

Supplement: Figure S4 — Evolutionary history inferred by using the Neighbor-Joining method based on 532 positions of pyrH gene sequence in the final dataset. The bootstrap test (1,000 replicates) are shown next to the branches. The evolutionary distances were computed using the Maximum Composite Likelihood method and are in the units of the number of base substitutions per site. The analysis involved 316 nucleotide sequences including only strains from AB region and type strains of each represented species. All ambiguous positions were removed for each sequence pair. White and red circles represent strains from benthic source and blue circles from pelagic. [file peerj-03-741-s004.png]

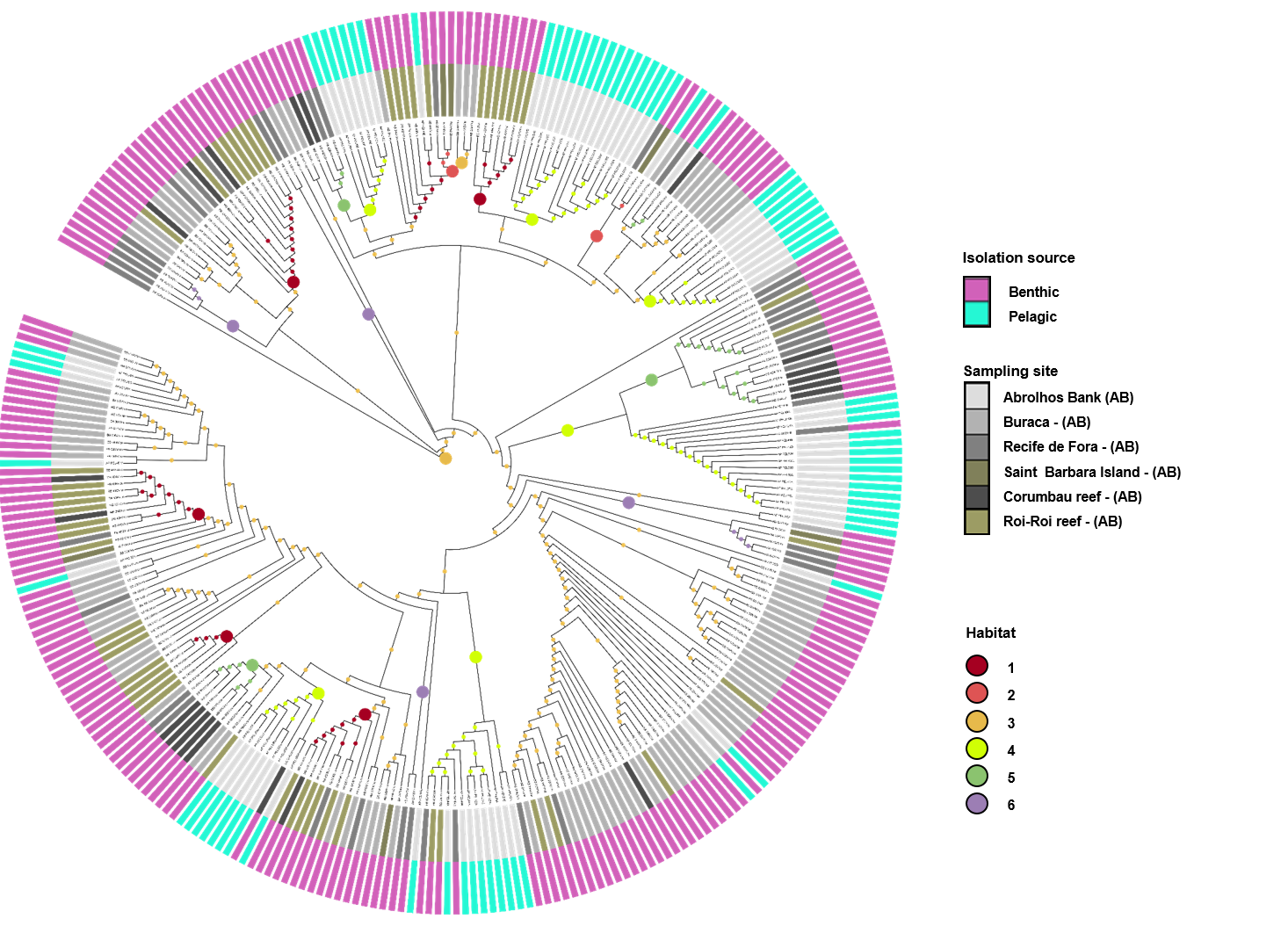

Supplement: Figure S5 — Inferred habitat associations for all ancestors of sequenced Vibrio strains from AB region. The rings surrounding the tree represent the isolation source (outer) and the collection point (inner) from which strains were isolated. The maximum likelihood assignment of nodes to habitats is shown for The maximum likelihood assignment of nodes to habitats is shown for clades supported by bootstraps >80%. Colored circles on each branch indicate the habitat assignment (H1-H6). Branch lengths were adjusted to aid visualization and do not represent evolutionary distances. [file peerj-03-741-s005.png]

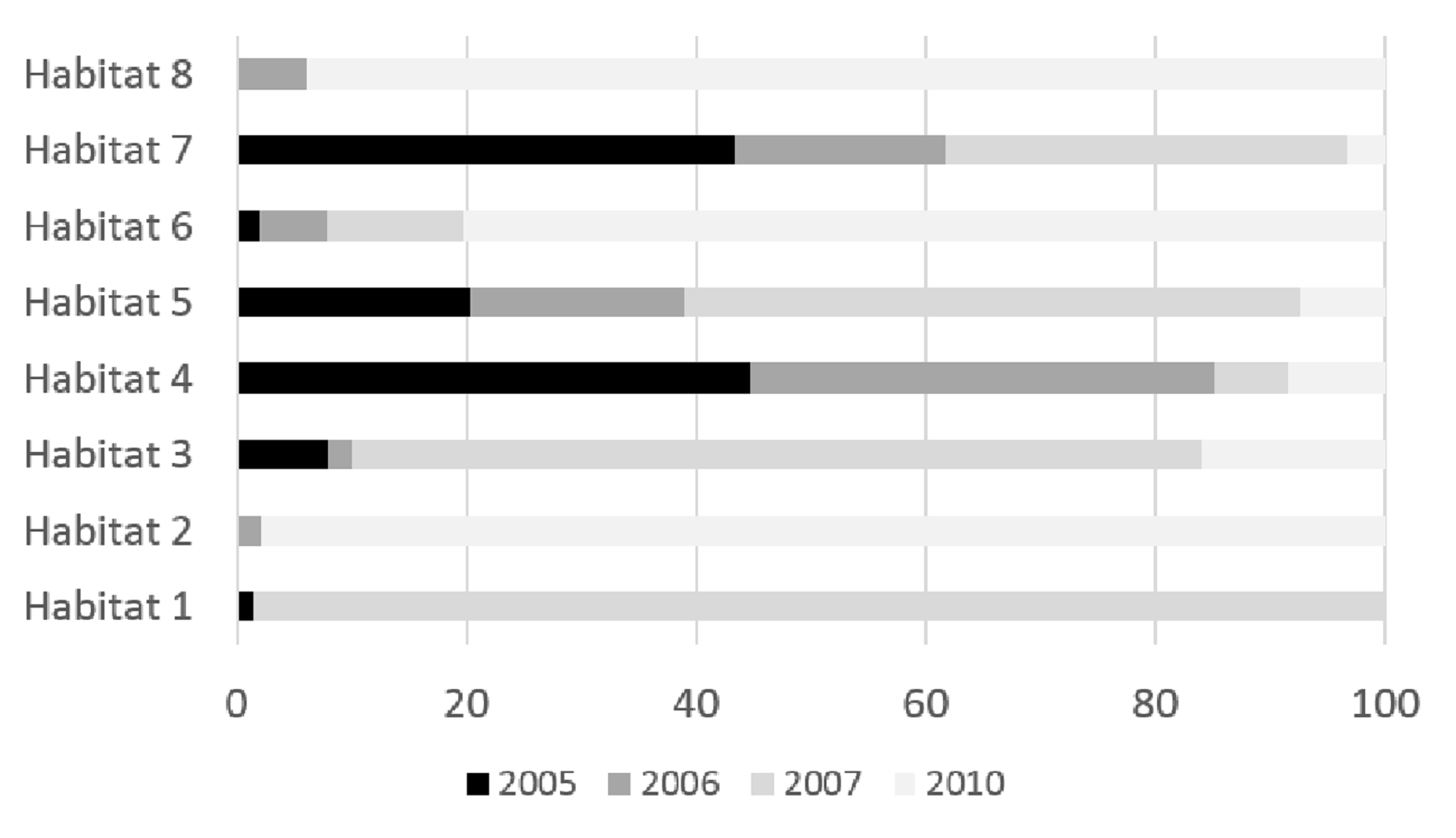

Supplement: Figure S6 — Habitat distribution according to sampling time. The values represents percentages. [file peerj-03-741-s006.png]
