# Supplementary material for: Diversity and ecological structure of vibrios in benthic and pelagic habitats along a latitudinal gradient in the Southwest Atlantic Ocean
Source: PeerJ. 2015 Feb 10;3:e741. doi: 10.7717/peerj.741 (PMC4327252; doi:10.7717/peerj.741)
Supplement: Table S1 [file peerj-03-741-s007.docx]

| **Collection Point** | **Isolation source** | **Collection date** | **Bacterial colony code** | **Identification** |
| --- | --- | --- | --- | --- |
| **Corumbau_Abrolhos Bank** | *Mussismilia hispida* | April 04, 2007 | 12K | *V. communis* |
|  | *Mussismilia hispida* | April 05, 2007 | 27A | *V. alginolyticus* |
|  | *Mussismilia hispida* | April 05, 2007 | 27B | *V. alginolyticus* |
|  | *Mussismilia hispida* | April 05, 2007 | 27C | *V. alginolyticus* |
|  | *Mussismilia hispida* | April 05, 2007 | 27D | *V. alginolyticus* |
|  | *Mussismilia hispida* | April 05, 2007 | 27E | *V. alginolyticus* |
|  | *Mussismilia hispida* | April 05, 2007 | 28A2 | *V. pelagius* |
|  | *Mussismilia hispida* | April 04, 2007 | 35A | *V. communis* |
|  | *Mussismilia hispida* | April 04, 2007 | 35B | *V. communis* |
|  | *Mussismilia braziliensis* | April 04, 2007 | 45A | *V. brasiliensis* |
|  | *Mussismilia braziliensis* | April 04, 2007 | 45B | *V. coralliilyticus* |
|  | *Mussismilia braziliensis* | April 04, 2007 | 45C | *V. coralliilyticus* |
|  | *Mussismilia braziliensis* | April 04, 2007 | 45D | *V. coralliilyticus* |
|  | *Mussismilia braziliensis* | April 04, 2007 | 45E | *V. brasiliensis* |
|  | *Mussismilia hispida* | April 04, 2007 | 50A | *V. communis* |
|  | *Mussismilia hispida* | April 04, 2007 | 50B | *V. communis* |
|  | *Mussismilia hispida* | April 04, 2007 | 50C | *V. communis* |
|  | *Mussismilia hispida* | April 04, 2007 | 50D | *V. communis* |
|  | *Mussismilia hispida* | April 04, 2007 | 50E | *V. harveyi* |
|  | *Mussismilia hispida* | April 04, 2007 | 50F | *V. communis* |
|  | *Mussismilia hispida* | April 04, 2007 | 50G | *V. communis* |
| **Roi-Roi_Abrolhos Bank** | *Mussismilia braziliensis* | April 06, 2007 | 38A | *V. coralliilyticus* |
|  | *Mussismilia braziliensis* | April 06, 2007 | 38B | *V. communis* |
|  | *Mussismilia braziliensis* | April 06, 2007 | 38C | *V. campbellii* |
|  | *Mussismilia braziliensis* | April 06, 2007 | 40B | *V. alginolyticus* |
|  | *Mussismilia braziliensis* | April 06, 2007 | 40E | *V. alginolyticus* |
|  | *Mussismilia hispida* | April 06, 2007 | 42A | *V. campbellii* |
|  | *Mussismilia hispida* | April 06, 2007 | 42B | *V. coralliilyticus* |
|  | *Mussismilia braziliensis* | April 06, 2007 | 43B | *V. coralliilyticus* |
|  | *Mussismilia braziliensis* | April 06, 2007 | 43C | *V. coralliilyticus* |
|  | *Mussismilia braziliensis* | April 06, 2007 | 43E | *V. communis* |
|  | *Mussismilia braziliensis* | April 06, 2007 | 43F | *V. communis* |
|  | *Mussismilia braziliensis* | April 06, 2007 | 43G | *V. communis* |
|  | *Mussismilia braziliensis* | April 06, 2007 | 43H | *V. communis* |
|  | *Mussismilia braziliensis* | April 06, 2007 | 43I | *V. tubiashii* |
|  | *Mussismilia braziliensis* | April 06, 2007 | 43K | *V. tubiashii* |
|  | *Mussismilia braziliensis* | April 06, 2007 | 43L | *V. communis* |
|  | *Mussismilia braziliensis* | April 06, 2007 | 43M | *V. communis* |
|  | *Mussismilia braziliensis* | April 06, 2007 | 43N | *V. communis* |
|  | *Mussismilia braziliensis* | April 06, 2007 | 43O | *V. coralliilyticus* |
|  | *Mussismilia braziliensis* | April 06, 2007 | 43P | *V. coralliilyticus* |
|  | *Mussismilia braziliensis* | April 06, 2007 | 43Q | *V. coralliilyticus* |
|  | *Mussismilia braziliensis* | April 06, 2007 | 43R | *V. communis* |
|  | *Mussismilia braziliensis* | April 06, 2007 | 43S | *V. communis* |
|  | *Mussismilia braziliensis* | April 06, 2007 | 43T | *V. communis* |
|  | *Mussismilia braziliensis* | April 06, 2007 | 43U | *V. sinaloensis* |
|  | *Mussismilia braziliensis* | April 06, 2007 | 43V | *V. sinaloensis* |
|  | *Mussismilia braziliensis* | April 06, 2007 | 43X | *V. sinaloensis* |
|  | *Mussismilia braziliensis* | April 06, 2007 | 43Y | *V. sinaloensis* |
|  | *Mussismilia braziliensis* | April 06, 2007 | 43Z | *V. tubiashii* |
|  | *Mussismilia braziliensis* | April 06, 2007 | 43AA | *V. communis* |
|  | *Mussismilia braziliensis* | April 06, 2007 | 43AB | *V. communis* |
|  | *Mussismilia braziliensis* | April 06, 2007 | 43AC | *V. coralliilyticus* |
|  | *Mussismilia braziliensis* | April 06, 2007 | 43AD | *V. sinaloensis* |
|  | *Mussismilia braziliensis* | April 06, 2007 | 43AF | *V. communis* |
|  | *Mussismilia braziliensis* | April 06, 2007 | 43AG | *V. communis* |
|  | *Mussismilia braziliensis* | April 06, 2007 | 43AH | *V. sinaloensis* |
|  | *Mussismilia braziliensis* | April 06, 2007 | 43AM | *V. sinaloensis* |
|  | *Mussismilia braziliensis* | April 06, 2007 | 74B | *V. communis* |
|  | *Mussismilia braziliensis* | April 06, 2007 | 74C | *V. communis* |
|  | *Mussismilia braziliensis* | April 06, 2007 | 74D | *V. communis* |
|  | *Mussismilia braziliensis* | April 06, 2007 | 74E | *V. rotiferianus* |
|  | *Mussismilia braziliensis* | April 06, 2007 | 74F | *V. harveyi* |
|  | *Mussismilia braziliensis* | April 06, 2007 | 74G | *V. harveyi* |
|  | *Mussismilia braziliensis* | April 06, 2007 | 74H | *V. harveyi* |
|  | *Mussismilia braziliensis* | April 06, 2007 | 74I | *V. harveyi* |
|  | *Mussismilia braziliensis* | April 06, 2007 | 74J | *V. tubiashi* |
|  | *Mussismilia braziliensis* | April 06, 2007 | 74K | *V. tubiashi* |
|  | *Mussismilia braziliensis* | April 06, 2007 | 74M | *V. communis* |
|  | *Mussismilia braziliensis* | April 06, 2007 | 74N | *V. communis* |
|  | *Mussismilia braziliensis* | April 06, 2007 | 74O | *V. communis* |
|  | *Mussismilia braziliensis* | April 06, 2007 | 74P | *V. communis* |
|  | *Mussismilia braziliensis* | April 06, 2007 | 74Q | *V. communis* |
| **Saint Barbara Island_Abrolhos Bank** | *Mussismilia braziliensis* | October 06, 2007 | PA1 | *V. rotiferianus* |
|  | *Mussismilia braziliensis* | October 06, 2007 | PA2 | *V.harveyi like* |
|  | *Mussismilia braziliensis* | October 06, 2007 | PA3 | *V.harveyi like* |
|  | *Mussismilia braziliensis* | October 06, 2007 | PA4 | *V. tubiashii* |
|  | *Mussismilia braziliensis* | October 07, 2007 | PA9 | *V. tubiashii* |
|  | *Mussismilia braziliensis* | October 07, 2007 | PA10 | *V. chagasii* |
| **Recife de For a_Abrolhos Bank** | *Phyllogorgia dilatata* | April 10, 2007 | 1S4 | *V. alginolyticus* |
|  | *Phyllogorgia dilatata* | April 10, 2007 | 3S5 | *V. rotiferianus* |
|  | *Phyllogorgia dilatata* | April 10, 2007 | 4S1 | *V. rotiferianus* |
|  | *Phyllogorgia dilatata* | April 10, 2007 | 4S2 | *V. rotiferianus* |
|  | *Phyllogorgia dilatata* | April 10, 2007 | 4S4 | *V. alginolyticus* |
|  | *Phyllogorgia dilatata* | April 10, 2007 | 1SA1 | *V. alginolyticus* |
|  | *Phyllogorgia dilatata* | April 10, 2007 | 1SA2 | *V. alginolyticus* |
|  | *Phyllogorgia dilatata* | April 10, 2007 | 2SA1 | *V. alginolyticus* |
|  | *Phyllogorgia dilatata* | April 10, 2007 | 2SA4 | *V. campbellii* |
|  | *Phyllogorgia dilatata* | April 10, 2007 | 2SA6 | *V. communis* |
|  | *Phyllogorgia dilatata* | April 10, 2007 | 2SA7 | *V. communis* |
|  | *Phyllogorgia dilatata* | April 10, 2007 | 3SA4 | *V. alginolyticus* |
|  | *Phyllogorgia dilatata* | April 10, 2007 | 3SA5 | *V. alginolyticus* |
|  | *Phyllogorgia dilatata* | April 10, 2007 | 4SA1 | *V*. sp. |
|  | *Phyllogorgia dilatata* | April 10, 2007 | 4SA2 | *V. communis* |
|  | *Phyllogorgia dilatata* | April 10, 2007 | 4SA3 | *V. communis* |
|  | *Phyllogorgia dilatata* | April 10, 2007 | 4SA4 | *V. brasiliensis* |
|  | *Phyllogorgia dilatata* | April 10, 2007 | 4SA5 | *V. coralliilyticus* |
|  | *Phyllogorgia dilatata* | April 10, 2007 | 4SA6 | *V. campbellii* |
|  | *Phyllogorgia dilatata* | April 10, 2007 | 4SA7 | *V. alginolyticus* |
|  | *Phyllogorgia dilatata* | April 10, 2007 | 4SA8 | *V. brasiliensis* |
|  | *Phyllogorgia dilatata* | April 10, 2007 | 1D1 | *V. diabolicus* |
|  | *Phyllogorgia dilatata* | April 10, 2007 | 1D2 | *V. xuii* |
|  | *Phyllogorgia dilatata* | April 10, 2007 | 1D3 | *V. alginolyticus* |
|  | *Phyllogorgia dilatata* | April 10, 2007 | 1D5 | *V. ponticus* |
|  | *Phyllogorgia dilatata* | April 10, 2007 | 2D2 | *V. nereis* |
|  | *Phyllogorgia dilatata* | April 10, 2007 | 3D1 | *V. communis* |
|  | *Phyllogorgia dilatata* | April 10, 2007 | 3D3 | *V. communis* |
|  | *Phyllogorgia dilatata* | April 10, 2007 | 4D2 | *V. diabolicus* |
|  | *Phyllogorgia dilatata* | April 10, 2007 | 4D3 | *V. communis* |
|  | *Phyllogorgia dilatata* | April 10, 2007 | 1DA1 | *V. chagasii* |
|  | *Phyllogorgia dilatata* | April 10, 2007 | 1DA2 | *V. pelagius* |
|  | *Phyllogorgia dilatata* | April 10, 2007 | 1DA3 | *V. communis* |
|  | *Phyllogorgia dilatata* | April 10, 2007 | 1DA4 | *V. tubiashii* |
|  | *Phyllogorgia dilatata* | April 10, 2007 | 1DA5 | *V. harveyi* |
|  | *Phyllogorgia dilatata* | April 10, 2007 | 2DA1 | *V. harveyi* |
|  | *Phyllogorgia dilatata* | April 10, 2007 | 2DA3 | *V. coralliilyticus* |
|  | *Phyllogorgia dilatata* | April 10, 2007 | 3DA1 | *V. coralliilyticus* |
|  | *Phyllogorgia dilatata* | April 10, 2007 | 3DA2 | *V. coralliilyticus* |
|  | *Phyllogorgia dilatata* | April 10, 2007 | 3DA3 | *V. coralliilyticus* |
|  | *Phyllogorgia dilatata* | April 10, 2007 | 3DA5 | *V. coralliilyticus* |
| **Buracas_ Abrolhos Bank** | Rhodolith (27m deep) | December 7, 2010 | G1 | *V. communis* |
|  | Rhodolith (27m deep) | December 7, 2010 | G2 | *V. harveyi* |
|  | Rhodolith (27m deep) | December 7, 2010 | G3 | *V. communis* |
|  | Rhodolith (27m deep) | December 7, 2010 | G4 | *V. communis* |
|  | Rhodolith (27m deep) | December 7, 2010 | G5 | *V. harveyi* |
|  | Rhodolith (43m deep) | December 5, 2010 | G7 | *V. harveyi* |
|  | Rhodolith (43m deep) | December 5, 2010 | G8 | *V. harveyi* |
|  | Rhodolith (43m deep) | December 5, 2010 | G9 | *V. communis* |
|  | Rhodolith (43m deep) | December 5, 2010 | G11 | *V. communis* |
|  | Rhodolith (43m deep) | December 5, 2010 | G12 | *V. communis* |
|  | Rhodolith (43m deep) | December 5, 2010 | G13 | *V. harveyi* |
|  | Rhodolith (43m deep) | December 5, 2010 | G14 | *V. communis* |
|  | Rhodolith (43m deep) | December 5, 2010 | G15 | candidate sp nov |
|  | Rhodolith (43m deep) | December 5, 2010 | G16 | *V. harveyi* |
|  | Rhodolith (43m deep) | December 5, 2010 | G17 | *V. harveyi* |
|  | Rhodolith (43m deep) | December 5, 2010 | G19 | *V. corallilyticus* |
|  | Rhodolith (43m deep) | December 5, 2010 | G20 | *V. communis* |
|  | Rhodolith (27m deep) | December 5, 2010 | G21 | *V. harveyi* |
|  | Rhodolith (27m deep) | December 7, 2010 | G22 | *V. harveyi* |
|  | Rhodolith (27m deep) | December 7, 2010 | G23 | *V. harveyi* |
|  | Rhodolith (27m deep) | December 7, 2010 | G24 | *V. harveyi* |
|  | Rhodolith (27m deep) | December 7, 2010 | G25 | *V. harveyi* |
|  | Rhodolith (27m deep) | December 7, 2010 | G26 | *V. harveyi* |
|  | Rhodolith (43m deep) | December 5, 2010 | G27 | *V. harveyi* |
|  | Rhodolith (43m deep) | December 5, 2010 | G28 | *V. harveyi* |
|  | Rhodolith (43m deep) | December 5, 2010 | G30 | *V. harveyi* |
|  | Rhodolith (43m deep) | December 5, 2010 | G31 | *V. harveyi* |
|  | Rhodolith (43m deep) | December 5, 2010 | G32 | *V. harveyi* |
|  | Rhodolith (43m deep) | December 5, 2010 | G33 | *V. harveyi* |
|  | Rhodolith (27m deep) | December 7, 2010 | G34 | *V. harveyi* |
|  | Rhodolith (27m deep) | December 7, 2010 | G35 | *V. communis* |
|  | Rhodolith (27m deep) | December 7, 2010 | G36 | *V. harveyi* |
|  | Rhodolith (43m deep) | December 7, 2010 | G37 | *V. harveyi* |
|  | Rhodolith (43m deep) | December 5, 2010 | G38 | *V. communis* |
|  | Rhodolith (43m deep) | December 5, 2010 | G39 | *V. harveyi* |
|  | Rhodolith (43m deep) | December 5, 2010 | G40 | *V. harveyi* |
|  | Rhodolith (43m deep) | December 5, 2010 | G41 | *V. harveyi* |
|  | Rhodolith (43m deep) | December 5, 2010 | G42 | *V. harveyi* |
|  | Rhodolith (43m deep) | December 5, 2010 | G43 | *V. harveyi* |
|  | Rhodolith (43m deep) | December 5, 2010 | G44 | candidate sp nov |
|  | Rhodolith (43m deep) | December 5, 2010 | G45 | *V. harveyi* |
|  | Rhodolith (43m deep) | December 5, 2010 | G46 | *V. harveyi* |
|  | Rhodolith (43m deep) | December 5, 2010 | G47 | candidate sp nov |
|  | Rhodolith (43m deep) | December 5, 2010 | G48 | *V. harveyi* |
|  | Rhodolith (43m deep) | December 5, 2010 | G49 | *V. harveyi* |
|  | Rhodolith (27m deep) | December 7, 2010 | G50 | *V. communis* |
|  | Rhodolith (27m deep) | December 7, 2010 | G51 | *V. communis* |
|  | Rhodolith (27m deep) | December 7, 2010 | G52 | *V. communis* |
|  | Rhodolith (27m deep) | December 7, 2010 | G53 | *V. communis* |
|  | Rhodolith (27m deep) | December 7, 2010 | G54 | *V. communis* |
|  | Rhodolith (27m deep) | December 7, 2010 | G55 | *V. tubiashi* |
|  | Rhodolith (27m deep) | December 7, 2010 | G57 | *V. communis* |
|  | Rhodolith (27m deep) | December 7, 2010 | G58 | candidate sp nov |
|  | Rhodolith (27m deep) | December 7, 2010 | G59 | candidate sp nov |
|  | Rhodolith (27m deep) | December 7, 2010 | G60 | *V. communis* |
|  | Rhodolith (27m deep) | December 7, 2010 | G61 | *V. communis* |
|  | Rhodolith (27m deep) | December 7, 2010 | G65 | candidate sp nov |
|  | Rhodolith (27m deep) | December 7, 2010 | G66 | candidate sp nov |
|  | Rhodolith (27m deep) | December 7, 2010 | G67 | *V. communis* |
|  | Rhodolith (27m deep) | December 7, 2010 | G68 | *V. communis* |
|  | Rhodolith (27m deep) | December 7, 2010 | G69 | candidate sp nov |
|  | Rhodolith (43m deep) | December 5, 2010 | G70 | *V. communis* |
|  | Rhodolith (43m deep) | December 5, 2010 | G72 | *V. communis* |
|  | Rhodolith (43m deep) | December 5, 2010 | G74 | candidate sp nov |
|  | Rhodolith (43m deep) | December 5, 2010 | G75 | candidate sp nov |
|  | Rhodolith (43m deep) | December 5, 2010 | G77 | candidate sp nov |
|  | Rhodolith (43m deep) | December 5, 2010 | G78 | *V. communis* |
|  | Rhodolith (43m deep) | December 5, 2010 | G79 | *V. communis* |
|  | Rhodolith (43m deep) | December 5, 2010 | G82 | *V. harveyi* |
|  | Rhodolith (43m deep) | December 6, 2010 | G83 | *V. harveyi* |
|  | Rhodolith (43m deep) | December 5, 2010 | G84 | *V. harveyi* |
|  | Rhodolith (43m deep) | December 5, 2010 | G85 | *V. harveyi* |
|  | Rhodolith (43m deep) | December 5, 2010 | G86 | *V. harveyi* |
|  | Rhodolith (43m deep) | December 5, 2010 | G91 | *V. tubiashi* |
|  | Rhodolith (43m deep) | December 5, 2010 | G92 | *V. tubiashi* |
|  | Rhodolith (43m deep) | December 5, 2010 | G95 | *V. communis* |
|  | Sediment | December 5, 2010 | 1 | *V. communis* |
|  | Sediment | December 5, 2010 | 2 | *V. communis* |
|  | Sediment | December 5, 2010 | 3 | *V. coralliilyticus* |
|  | Sediment | December 5, 2010 | 7 | *V. coralliilyticus* |
|  | Sediment | December 5, 2010 | 11 | *V. agarivorans* |
|  | Sediment | December 5, 2010 | 12 | *V. coralliilyticus* |
|  | Sediment | December 5, 2010 | 15 | *V. pelagius* |
|  | Sediment | December 5, 2010 | 18 | *V. coralliilyticus* |
|  | Sediment | December 5, 2010 | 20 | *V. harveyi* |
|  | Sediment | December 5, 2010 | 21 | *V. communis* |
|  | Sediment | December 5, 2010 | 22 | *V. communis* |
|  | Sediment | December 5, 2010 | 23 | *V. communis* |
|  | Sediment | December 5, 2010 | 24 | *V. harveyi* |
|  | Sediment | December 5, 2010 | 25 | *V. harveyi* |
|  | Sediment | December 5, 2010 | 26 | *V. harveyi* |
|  | Sediment | December 5, 2010 | 27 | *V. communis* |
|  | Sediment | December 5, 2010 | 28 | *V. harveyi* |
|  | Sediment | December 5, 2010 | 29 | *V. harveyi* |
|  | Sediment | December 5, 2010 | 30 | *V. communis* |
|  | Sediment | December 5, 2010 | 32 | *V. harveyi* |
|  | Sediment | December 5, 2010 | 33 | *V. harveyi* |
|  | Sediment | December 5, 2010 | 34 | *V. coralliilyticus* |
|  | Sediment | December 5, 2010 | 35 | *V.* sp*.* |
|  | Sediment | December 5, 2010 | 37 | *V. pelagius* |
|  | Sediment | December 5, 2010 | 38 | *V. coralliilyticus* |
| **Abrolhos Bank** | Water (station 65 : 150m deep) | July 28, 2007 | PEL4D | *V.communis* |
|  | Water (station 61 :10m deep) | July 31, 2007 | PEL20A | *V.hepatarius* |
|  | Water (station 61 :10m deep) | July 31, 2007 | PEL20B | *V.hepatarius* |
|  | Water (station 61 :10m deep) | July 31, 2007 | PEL20C | *V.hepatarius* |
|  | Water (station 61 :10m deep) | July 31, 2007 | PEL20E | *V.hepatarius* |
|  | Water (station 61 :10m deep) | July 31, 2007 | PEL20F | *V.hepatarius* |
|  | Water (station 61 :10m deep) | July 31, 2007 | PEL20G | *V.hepatarius* |
|  | Water (station 61 :10m deep) | July 31, 2007 | PEL20H | *V.hepatarius* |
|  | Water (station 61 :10m deep) | July 31, 2007 | PEL21A | *V.maritimus group* |
|  | Water (station 61 :10m deep) | July 31, 2007 | PEL21B | *V.maritimus group* |
|  | Water (station 61 :10m deep) | July 31, 2007 | PEL21C | *V.maritimus group* |
|  | Water (station 61 :10m deep) | July 31, 2007 | PEL21D | *V.maritimus group* |
|  | Water (station 61 :10m deep) | July 31, 2007 | PEL21E | *V.maritimus group* |
|  | Water (station 61 :10m deep) | July 31, 2007 | PEL21F | *V.maritimus group* |
|  | Water (station 61 :10m deep) | July 31, 2007 | PEL21G | *V.maritimus group* |
|  | Water (station 61 :10m deep) | July 31, 2007 | PEL22A | *V.campbellii* |
|  | Water (station 61 :10m deep) | July 31, 2007 | PEL22B | *V.pelagius* |
|  | Water (station 61 :10m deep) | July 31, 2007 | PEL22C | *V.campbellii* |
|  | Water (station 61 :10m deep) | July 31, 2007 | PEL23A | *V.campbellii* |
|  | Water (station 61 :10m deep) | July 31, 2007 | PEL23B | *V.campbellii* |
|  | Water (station 61 :10m deep) | July 31, 2007 | PEL23C | *V.campbellii* |
|  | Water (station 61 :10m deep) | July 31, 2007 | PEL23D | *V.campbellii* |
|  | Water (station 61 :10m deep) | July 31, 2007 | PEL26D | *V.communis* |
|  | Water (station 61 :10m deep) | July 31, 2007 | PEL26E | *V.communis* |
|  | Water (station 61 :10m deep) | July 31, 2007 | PEL26G | *V.communis* |
|  | Water (station 65 :10m deep) | July 28, 2007 | PEL36A | *V.communis* |
|  | Water (station 65 : 10m deep) | July 28, 2007 | PEL36B | *V.harveyi* |
|  | Water (station 65 : 10m deep) | July 28, 2007 | PEL36D | *V.harveyi* |
|  | Water (station 65 : 10m deep) | July 28, 2007 | PEL36G | *V.harveyi* |
|  | Water (station 65 : 10m deep) | July 28, 2007 | PEL37A | *V.diabolicus* |
|  | Water (station 65 : 10m deep) | July 28, 2007 | PEL37B | *V.diabolicus* |
|  | Water (station 65 : 10m deep) | July 28, 2007 | PEL37C | *V.diabolicus* |
|  | Water (station 65 : 75m deep) | July 28, 2007 | PEL38A | *V.diabolicus* |
|  | Water (station 65 : 75m deep) | July 28, 2007 | PEL38B | *V.diabolicus* |
|  | Water (station 65 : 75m deep) | July 28, 2007 | PEL38C | *V.diabolicus* |
|  | Water (station 65 : 150m deep) | July 28, 2007 | PEL40B | *V.diabolicus* |
|  | Water (station 65 : 150m deep) | July 28, 2007 | PEL40C | *V.diabolicus* |
|  | Water (station 65 : 150m deep) | July 28, 2007 | PEL40D | *V.diabolicus* |
|  | Water (station 65 : 150m deep) | July 28, 2007 | PEL40E | *V.diabolicus* |
|  | Water (station 65 : 150m deep) | July 28, 2007 | PEL41B | *V.diabolicus* |
|  | Water (station 65 : 150m deep) | July 28, 2007 | PEL41D | *V.diabolicus* |
|  | Water (station 61 :10m deep) | July 31, 2007 | PEL44A | *V.campbellii* |
|  | Water (station 61 :10m deep) | July 31, 2007 | PEL45A | *V.campbellii* |
|  | Water (station 65 : 75m deep) | July 28, 2007 | PEL47A | *V.chagasii* |
|  | Water (station 65 : 75m deep) | July 28, 2007 | PEL48A | *V.communis* |
|  | Water (station 65 : 75m deep) | July 28, 2007 | PEL48B | *V.campbellii* |
|  | Water (station 65 : 75m deep) | July 28, 2007 | PEL68D | *V.tubiashii* |
|  | Water (station 61 :10m deep) | July 31, 2007 | PEL102A | *V.maritimus* |
|  | Water (station 61 :10m deep) | July 31, 2007 | PEL102B | *V.maritimus* |
|  | Water (station 61 :10m deep) | July 31, 2007 | PEL103A | *V.communis* |
|  | Water (station 61 :10m deep) | July 31, 2007 | PEL104A | *V.communis* |
|  | Water (station 61 :10m deep) | July 31, 2007 | PEL105A | *V.communis* |
|  | Water (station 61 :10m deep) | July 31, 2007 | PEL106A | *V.maritimus* |
|  | Water (station 61 :10m deep) | July 31, 2007 | PEL107A | *V.communis* |
|  | Water (station 61 :10m deep) | July 31, 2007 | PEL108A | *V.communis* |
|  | Water (station 61 :10m deep) | July 31, 2007 | PEL109A | *V.communis* |
|  | Water (station 61 :10m deep) | July 31, 2007 | PEL110A | *V.communis* |
|  | Water (station 61 :10m deep) | July 31, 2007 | PEL111A | *V.maritimus* |
|  | Water (station 61 :10m deep) | July 31, 2007 | PEL112A | *V.communis* |
|  | Water (station 65 : 150m deep) | July 28, 2007 | PEL115A | *V.pelagius* |
|  | Water (station 65 : 150m deep) | July 28, 2007 | PEL115B | *V.pelagius* |
|  | Water (station 65 : 150m deep) | July 28, 2007 | PEL115C | *V.pelagius* |
|  | Water (station 65 : 150m deep) | July 28, 2007 | PEL115D | *V.pelagius* |
|  | Water (station 65 : 150m deep) | July 28, 2007 | PEL115E | *V.pelagius* |
|  | Water (station 65 : 150m deep) | July 28, 2007 | PEL115F | *V.pelagius* |
|  | Water (station 65 : 150m deep) | July 28, 2007 | PEL115G | *V.pelagius* |
|  | Water (station 65 : 150m deep) | July 28, 2007 | PEL115H | *V.pelagius* |
|  | Water (station 65 : 150m deep) | July 28, 2007 | PEL115J | *V.pelagius* |
|  | Water (station 65 : 150m deep) | July 28, 2007 | PEL118A | *V.diabolicus* |
|  | Water (station 65 : 150m deep) | July 28, 2007 | PEL118B | *V.diabolicus* |
|  | Water (station 65 : 75m deep) | July 28, 2007 | PEL119A | *V.alfacsensis* |
|  | Water (station 65 : 150m deep) | July 28, 2007 | PEL121C | *V.maritimus* |
|  | Water (station 61 :10m deep) | July 31, 2007 | PEL122A | *V.maritimus* |
|  | Water (station 61 :10m deep) | July 31, 2007 | PEL124A | *V.maritimus* |
|  | Water (station 61 :10m deep) | July 31, 2007 | PEL125A | *V.maritimus* |
|  | Water (station 61 :10m deep) | July 31, 2007 | PEL125B | *V.maritimus* |
| **St. Peter and St. Paul Archipelago** | *Madracis decactis* | Sep 22, 2010 | A-15 | *Vibrio tubiashii* |
|  | *Hermodice carunculata* | Sep 22, 2010 | A-16 | *V. shiloi* |
|  | *Hermodice carunculata* | Sep 22, 2010 | A-17 | *V. shiloi* |
|  | *Hermodice carunculata* | Sep 22, 2010 | A-18 | *V. shiloi* |
|  | *Hermodice carunculata* | Sep 22, 2010 | A-19 | *V. shiloi* |
|  | *Hermodice carunculata* | Sep 22, 2010 | A-20 | *V. shiloi* |
|  | *Hermodice carunculata* | Sep 22, 2010 | A-23 | *V. shiloi* |
|  | *Hermodice carunculata* | Sep 22, 2010 | A-24 | *V. shiloi* |
|  | *Hermodice carunculata* | Sep 22, 2010 | A-26 | *V. shiloi* |
|  | *Hermodice carunculata* | Sep 22, 2010 | A-28 | *V. shiloi* |
|  | *Hermodice carunculata* | Sep 22, 2010 | A-30 | *V. shiloi* |
|  | *Hermodice carunculata* | Sep 22, 2010 | A-31 | *V. shiloi* |
|  | *Hermodice carunculata* | Sep 22, 2010 | A-32 | *V. shiloi* |
|  | *Hermodice carunculata* | Sep 22, 2010 | A-33 | *V. shiloi* |
|  | *Madracis decactis* | Sep 22, 2010 | A-36 | *V. tubiashii* |
|  | *Scolymia wellsi* | Sep 14, 2010 | A-37 | *V.communis* |
|  | *Scolymia wellsi* | Sep 14, 2010 | A-38 | *V.communis* |
|  | *Scolymia wellsi* | Sep 14, 2010 | A-39 | *V.communis* |
|  | *Scolymia wellsi* | Sep 14, 2010 | A-40 | *V.communis* |
|  | *Scolymia wellsi* | Sep 14, 2010 | A-41 | *V.communis* |
|  | *Scolymia wellsi* | Sep 14, 2010 | A-42 | *V.communis* |
|  | *Scolymia wellsi* | Sep 14, 2010 | A-43 | *V.communis* |
|  | *Scolymia wellsi* | Sep 14, 2010 | A-44 | *V.communis* |
|  | *Scolymia wellsi* | Sep 14, 2010 | A-45 | *V.communis* |
|  | *Scolymia wellsi* | Sep 14, 2010 | A-46 | *V.communis* |
|  | *Scolymia wellsi* | Sep 14, 2010 | A-47 | *V.communis* |
|  | *Scolymia wellsi* | Sep 14, 2010 | A-51 | *V.communis* |
|  | *Scolymia wellsi* | Sep 14, 2010 | A-52 | *V.communis* |
|  | *Scolymia wellsi* | Sep 14, 2010 | A-53 | *V.communis* |
|  | *Scolymia wellsi* | Sep 14, 2010 | A-54 | *V.communis* |
|  | *Scolymia wellsi* | Sep 14, 2010 | A-55 | *V. harveyi* |
|  | *Scolymia wellsi* | Sep 14, 2010 | A-56 | *V. harveyi* |
|  | *Scolymia wellsi* | Sep 14, 2010 | A-57 | *V. harveyi* |
|  | *Hermodice carunculata* | Sep 22, 2010 | A-59 | *V. shiloi* |
|  | *Hermodice carunculata* | Sep 22, 2010 | A-60 | *V. shiloi* |
|  | *Hermodice carunculata* | Sep 22, 2010 | A-91 | *V. shiloi* |
|  | *Hermodice carunculata* | Sep 22, 2010 | A-92 | *V. shiloi* |
|  | *Hermodice carunculata* | Sep 22, 2010 | A-93 | *V. shiloi* |
|  | *Hermodice carunculata* | Sep 22, 2010 | A-94 | *V. shiloi* |
|  | *Madracis decactis* | Sep 14, 2010 | A-95 | *V. campbellii* |
|  | *Madracis decactis* | Sep 14, 2010 | A-96 | *Vibrio* sp. |
|  | *Madracis decactis* | Sep 14, 2010 | A-97 | *V. campbellii* |
|  | *Madracis decactis* | Sep 14, 2010 | A-98 | *V. campbellii* |
|  | *Hermodice carunculata* | Sep 22, 2010 | A-99 | *V. shiloi* |
|  | *Madracis decactis* | Sep 14, 2010 | A-100 | *V. campbellii* |
|  | *Madracis decactis* | Sep 14, 2010 | A-101 | *V. campbellii* |
|  | *Madracis decactis* | Sep 14, 2010 | A-102 | *V. campbellii* |
|  | *Madracis decactis* | Sep 14, 2010 | A-103 | *Vibrio* sp. |
|  | *Madracis decactis* | Sep 14, 2010 | A-104 | *V. campbellii* |
|  | *Madracis decactis* | Sep 14, 2010 | A-105 | *V. campbellii* |
|  | *Madracis decactis* | Sep 14, 2010 | A-106 | *V. maritimus* |
|  | *Madracis decactis* | Sep 14, 2010 | A-107 | *V. maritimus* |
|  | *Madracis decactis* | Sep 14, 2010 | A-108 | *V. campbellii* |
|  | *Madracis decactis* | Sep 14, 2010 | A-109 | *V. campbellii* |
|  | *Madracis decactis* | Sep 14, 2010 | A-110 | *Vibrio* sp. |
|  | *Madracis decactis* | Sep 14, 2010 | A-111 | *V. campbellii* |
|  | *Hermodice carunculata* | Sep 22, 2010 | A-112 | *V. shiloi* |
|  | *Hermodice carunculata* | Sep 22, 2010 | A-113 | *V. shiloi* |
|  | *Hermodice carunculata* | Sep 22, 2010 | A-114 | *V. shiloi* |
|  | *Madracis decactis* | Sep 14, 2010 | A-115 | *V. maritimus* |
|  | *Hermodice carunculata* | Sep 22, 2010 | A-116 | *V. shiloi* |
|  | *Hermodice carunculata* | Sep 22, 2010 | A-117 | *V. shiloi* |
|  | *Hermodice carunculata* | Sep 22, 2010 | A-118 | *V. shiloi* |
|  | *Hermodice carunculata* | Sep 22, 2010 | A-119 | *V. shiloi* |
|  | *Hermodice carunculata* | Sep 22, 2010 | A-120 | *V. shiloi* |
|  | *Hermodice carunculata* | Sep 22, 2010 | A-121 | *V. shiloi* |
|  | *Hermodice carunculata* | Sep 22, 2010 | A-122 | *V. shiloi* |
|  | *Hermodice carunculata* | Sep 22, 2010 | A-123 | *V. shiloi* |
|  | *Hermodice carunculata* | Sep 22, 2010 | A-124 | *V. shiloi* |
|  | *Hermodice carunculata* | Sep 22, 2010 | A-125 | *V. shiloi* |
|  | *Hermodice carunculata* | Sep 22, 2010 | A-126 | *V. shiloi* |
|  | *Hermodice carunculata* | Sep 22, 2010 | A-127 | *V. shiloi* |
|  | *Hermodice carunculata* | Sep 22, 2010 | A-128 | *V. shiloi* |
|  | *Hermodice carunculata* | Sep 22, 2010 | A-129 | *V. shiloi* |
|  | *Hermodice carunculata* | Sep 22, 2010 | A-130 | *V. shiloi* |
|  | *Hermodice carunculata* | Sep 22, 2010 | A-131 | *V. shiloi* |
|  | *Hermodice carunculata* | Sep 22, 2010 | A-132 | *V. shiloi* |
|  | *Hermodice carunculata* | Sep 22, 2010 | A-133 | *V. shiloi* |
|  | *Hermodice carunculata* | Sep 22, 2010 | A-134 | *V. shiloi* |
|  | *Hermodice carunculata* | Sep 22, 2010 | A-135 | *V. shiloi* |
|  | *Hermodice carunculata* | Sep 22, 2010 | A-136 | *V. shiloi* |
|  | *Hermodice carunculata* | Sep 22, 2010 | A-137 | *V. shiloi* |
|  | *Hermodice carunculata* | Sep 22, 2010 | A-138 | *V. shiloi* |
|  | *Hermodice carunculata* | Sep 22, 2010 | A-139 | *V. shiloi* |
|  | *Hermodice carunculata* | Sep 22, 2010 | A-140 | *V. shiloi* |
|  | *Hermodice carunculata* | Sep 22, 2010 | A-142 | *V. shiloi* |
|  | *Hermodice carunculata* | Sep 22, 2010 | A-143 | *V. shiloi* |
|  | *Hermodice carunculata* | Sep 22, 2010 | A-144 | *V. shiloi* |
|  | *Hermodice carunculata* | Sep 22, 2010 | A-145 | *V. shiloi* |
|  | *Hermodice carunculata* | Sep 22, 2010 | A-146 | *V. shiloi* |
|  | *Hermodice carunculata* | Sep 22, 2010 | A-147 | *V. shiloi* |
|  | *Hermodice carunculata* | Sep 22, 2010 | A-148 | *V. shiloi* |
|  | *Hermodice carunculata* | Sep 22, 2010 | A-149 | *V. shiloi* |
|  | *Hermodice carunculata* | Sep 22, 2010 | A-150 | *V. shiloi* |
|  | *Hermodice carunculata* | Sep 22, 2010 | A-151 | *V. shiloi* |
|  | *Hermodice carunculata* | Sep 22, 2010 | A-152 | *V. shiloi* |
|  | *Hermodice carunculata* | Sep 22, 2010 | A-153 | *V. shiloi* |
|  | *Hermodice carunculata* | Sep 22, 2010 | A-154 | *V. shiloi* |
|  | *Hermodice carunculata* | Sep 22, 2010 | A-155 | *V. shiloi* |
|  | *Hermodice carunculata* | Sep 22, 2010 | A-157 | *V. shiloi* |
|  | *Hermodice carunculata* | Sep 22, 2010 | A-158 | *V. shiloi* |
|  | *Hermodice carunculata* | Sep 22, 2010 | A-159 | *V. shiloi* |
|  | *Hermodice carunculata* | Sep 22, 2010 | A-160 | *V. shiloi* |
|  | *Hermodice carunculata* | Sep 22, 2010 | A-161 | *V. shiloi* |
|  | *Hermodice carunculata* | Sep 22, 2010 | A-162 | *V. shiloi* |
|  | *Hermodice carunculata* | Sep 22, 2010 | A-163 | *V. shiloi* |
|  | *Hermodice carunculata* | Sep 22, 2010 | A-164 | *V. shiloi* |
|  | *Hermodice carunculata* | Sep 22, 2010 | A-165 | *V. shiloi* |
|  | *Hermodice carunculata* | Sep 22, 2010 | A-166 | *V. shiloi* |
|  | *Hermodice carunculata* | Sep 22, 2010 | A-167 | *V. shiloi* |
|  | *Hermodice carunculata* | Sep 22, 2010 | A-168 | *V. shiloi* |
|  | *Hermodice carunculata* | Sep 22, 2010 | A-169 | *V. shiloi* |
|  | *Hermodice carunculata* | Sep 22, 2010 | A-170 | *V. shiloi* |
|  | *Hermodice carunculata* | Sep 22, 2010 | A-171 | *V. shiloi* |
|  | *Hermodice carunculata* | Sep 22, 2010 | A-172 | *V. shiloi* |
|  | *Hermodice carunculata* | Sep 22, 2010 | A-173 | *V. shiloi* |
|  | *Hermodice carunculata* | Sep 22, 2010 | A-174 | *V. shiloi* |
|  | *Hermodice carunculata* | Sep 22, 2010 | A-175 | *V. shiloi* |
|  | *Hermodice carunculata* | Sep 22, 2010 | A-176 | *V. shiloi* |
|  | *Hermodice carunculata* | Sep 22, 2010 | A-177 | *V. shiloi* |
|  | *Hermodice carunculata* | Sep 22, 2010 | A-178 | *V. shiloi* |
|  | *Hermodice carunculata* | Sep 22, 2010 | A-179 | *V. shiloi* |
|  | *Hermodice carunculata* | Sep 22, 2010 | A-180 | *V. shiloi* |
|  | *Hermodice carunculata* | Sep 22, 2010 | A-181 | *V. shiloi* |
|  | *Hermodice carunculata* | Sep 22, 2010 | A-182 | *V. shiloi* |
|  | *Hermodice carunculata* | Sep 22, 2010 | A-183 | *V. shiloi* |
|  | *Hermodice carunculata* | Sep 22, 2010 | A-184 | *V. shiloi* |
|  | *Hermodice carunculata* | Sep 22, 2010 | A-185 | *V. shiloi* |
|  | *Hermodice carunculata* | Sep 22, 2010 | A-192 | *V. shiloi* |
|  | *Hermodice carunculata* | Sep 22, 2010 | A-193 | *V. shiloi* |
|  | *Hermodice carunculata* | Sep 22, 2010 | A-194 | *V. shiloi* |
|  | *Hermodice carunculata* | Sep 22, 2010 | A-195 | *V. shiloi* |
|  | *Hermodice carunculata* | Sep 22, 2010 | A-196 | *V. shiloi* |
|  | *Hermodice carunculata* | Sep 22, 2010 | A-197 | *V. shiloi* |
|  | *Hermodice carunculata* | Sep 22, 2010 | A-198 | *V. shiloi* |
|  | *Hermodice carunculata* | Sep 22, 2010 | A-199 | *V. shiloi* |
|  | *Hermodice carunculata* | Sep 22, 2010 | A-200 | *V. shiloi* |
|  | *Hermodice carunculata* | Sep 22, 2010 | A-201 | *V. shiloi* |
|  | *Hermodice carunculata* | Sep 22, 2010 | A-202 | *V. shiloi* |
|  | *Hermodice carunculata* | Sep 22, 2010 | A-203 | *V. shiloi* |
|  | *Hermodice carunculata* | Sep 22, 2010 | A-204 | *V. shiloi* |
|  | *Hermodice carunculata* | Sep 22, 2010 | A-205 | *V. shiloi* |
|  | *Hermodice carunculata* | Sep 22, 2010 | A-206 | *V. shiloi* |
|  | *Hermodice carunculata* | Sep 22, 2010 | A-207 | *V. shiloi* |
|  | *Hermodice carunculata* | Sep 22, 2010 | A-208 | *V. shiloi* |
|  | *Hermodice carunculata* | Sep 22, 2010 | A-209 | *V. shiloi* |
|  | *Hermodice carunculata* | Sep 22, 2010 | A-210 | *V. shiloi* |
|  | *Hermodice carunculata* | Sep 22, 2010 | A-211 | *V. shiloi* |
|  | *Hermodice carunculata* | Sep 22, 2010 | A-212 | *V. shiloi* |
|  | *Hermodice carunculata* | Sep 22, 2010 | A-213 | *V. furnissii* |
|  | *Hermodice carunculata* | Sep 22, 2010 | A-214 | *V. shiloi* |
|  | *Hermodice carunculata* | Sep 22, 2010 | A-215 | *V. shiloi* |
|  | *Hermodice carunculata* | Sep 22, 2010 | A-216 | *V. shiloi* |
|  | *Hermodice carunculata* | Sep 22, 2010 | A-217 | *V. shiloi* |
|  | *Hermodice carunculata* | Sep 22, 2010 | A-218 | *V. shiloi* |
|  | *Hermodice carunculata* | Sep 22, 2010 | A-219 | *V. shiloi* |
|  | *Hermodice carunculata* | Sep 22, 2010 | A-220 | *V. shiloi* |
|  | *Hermodice carunculata* | Sep 22, 2010 | A-221 | *V. shiloi* |
|  | *Hermodice carunculata* | Sep 22, 2010 | A-222 | *V. furnissii* |
|  | *Hermodice carunculata* | Sep 22, 2010 | A-223 | *V. furnissii* |
|  | *Hermodice carunculata* | Sep 22, 2010 | A-224 | *V. shiloi* |
|  | *Hermodice carunculata* | Sep 22, 2010 | A-225 | *V. furnissii* |
|  | *Hermodice carunculata* | Sep 22, 2010 | A-226 | *V. shiloi* |
|  | *Hermodice carunculata* | Sep 22, 2010 | A-227 | *V. shiloi* |
|  | *Hermodice carunculata* | Sep 22, 2010 | A-228 | *V. shiloi* |
|  | *Hermodice carunculata* | Sep 22, 2010 | A-229 | *V. shiloi* |
|  | *Hermodice carunculata* | Sep 22, 2010 | A-230 | *V. shiloi* |
|  | *Hermodice carunculata* | Sep 22, 2010 | A-261 | *V. shiloi* |
|  | *Hermodice carunculata* | Sep 22, 2010 | A-263 | *V. shiloi* |
|  | *Hermodice carunculata* | Sep 22, 2010 | A-264 | *V. shiloi* |
|  | *Hermodice carunculata* | Sep 22, 2010 | A-265 | *V. shiloi* |
|  | *Hermodice carunculata* | Sep 22, 2010 | A-266 | *V. shiloi* |
|  | *Hermodice carunculata* | Sep 22, 2010 | A-271 | *V. shiloi* |
|  | *Hermodice carunculata* | Sep 22, 2010 | A-274 | *V. shiloi* |
|  | *Madracis decactis* | Sep 22, 2010 | A-290 | *V. communis* |
|  | *Madracis decactis* | Sep 22, 2010 | A-291 | *V. communis* |
|  | *Madracis decactis* | Sep 22, 2010 | A-292 | *V. communis* |
|  | *Madracis decactis* | Sep 22, 2010 | A-293 | *V. communis* |
|  | *Hermodice carunculata* | Sep 22, 2010 | A-294 | *V. shiloi* |
|  | *Madracis decactis* | Sep 14, 2010 | A-295 | *Vibrio* sp. |
|  | *Madracis decactis* | Sep 14, 2010 | A-296 | *V. campbellii* |
|  | *Madracis decactis* | Sep 14, 2010 | A-297 | *V. maritimus/V.variabilis group* |
|  | *Madracis decactis* | Sep 14, 2010 | A-298 | *V. communis* |
|  | *Madracis decactis* | Sep 14, 2010 | A-299 | *V. communis* |
|  | *Madracis decactis* | Sep 14, 2010 | A-300 | *V. communis* |
|  | *Madracis decactis* | Sep 14, 2010 | A-301 | *V. communis* |
|  | *Madracis decactis* | Sep 14, 2010 | A-302 | *Vibrio* sp. |
|  | *Madracis decactis* | Sep 14, 2010 | A-303 | *Vibrio* sp. |
|  | *Madracis decactis* | Sep 14, 2010 | A-304 | *V. harveyi* |
|  | *Madracis decactis* | Sep 14, 2010 | A-305 | *Vibrio* sp. |
|  | *Madracis decactis* | Sep 14, 2010 | A-306 | *Vibrio* sp. |
|  | *Madracis decactis* | Sep 14, 2010 | A-307 | *Vibrio* sp. |
|  | *Madracis decactis* | Sep 14, 2010 | A-308 | *V. harveyi* |
|  | *Madracis decactis* | Sep 14, 2010 | A-311 | *V. campbellii* |
|  | *Madracis decactis* | Sep 14, 2010 | A-312 | *Vibrio* sp. |
|  | *Madracis decactis* | Sep 14, 2010 | A-313 | *V. communis* |
|  | *Madracis decactis* | Sep 14, 2010 | A-314 | *Vibrio* sp. |
|  | *Madracis decactis* | Sep 14, 2010 | A-315 | *V. harveyi* |
|  | *Madracis decactis* | Sep 14, 2010 | A-316 | *V. harveyi* |
|  | *Madracis decactis* | Sep 14, 2010 | A-317 | *V. harveyi* |
|  | *Madracis decactis* | Sep 14, 2010 | A-318 | *V. campbellii* |
|  | *Hermodice carunculata* | Sep 22, 2010 | A-319 | *V. shiloi* |
|  | *Madracis decactis* | Sep 14, 2010 | A-320 | *V. maritimus/V.variabilis group* |
|  | *Madracis decactis* | Sep 14, 2010 | A-321 | *V. maritimus/V.variabilis group* |
|  | *Madracis decactis* | Sep 14, 2010 | A-322 | *V. maritimus/V.variabilis group* |
|  | *Madracis decactis* | Sep 14, 2010 | A-323 | *V. pelagius* |
|  | *Madracis decactis* | Sep 14, 2010 | A-324 | *V. pelagius* |
|  | *Madracis decactis* | Sep 14, 2010 | A-325 | *V. pelagius* |
|  | *Madracis decactis* | Sep 14, 2010 | A-326 | *Vibrio* sp*.* nov. |
|  | *Madracis decactis* | Sep 14, 2010 | A-327 | *V. maritimus* |
|  | *Madracis decactis* | Sep 14, 2010 | A-328 | *V. madracius* |
|  | *Madracis decactis* | Sep 14, 2010 | A-329 | *V. pelagius* |
|  | *Madracis decactis* | Sep 14, 2010 | A-330 | *V. campbellii* |
|  | *Madracis decactis* | Sep 14, 2010 | A-331 | *V. communis* |
|  | *Madracis decactis* | Sep 14, 2010 | A-332 | *V. communis* |
|  | *Madracis decactis* | Sep 14, 2010 | A-333 | *V. campbellii* |
|  | *Madracis decactis* | Sep 14, 2010 | A-334 | *V. campbellii* |
|  | *Madracis decactis* | Sep 14, 2010 | A-335 | *V. communis* |
|  | *Madracis decactis* | Sep 14, 2010 | A-336 | *V. communis* |
|  | *Madracis decactis* | Sep 14, 2010 | A-337 | *V. communis* |
|  | *Madracis decactis* | Sep 14, 2010 | A-338 | *V. maritimus/V.variabilis group* |
|  | *Madracis decactis* | Sep 14, 2010 | A-340 | *V. maritimus* |
|  | *Madracis decactis* | Sep 14, 2010 | A-341 | *V. campbellii* |
|  | *Madracis decactis* | Sep 14, 2010 | A-342 | *V. campbellii* |
|  | *Madracis decactis* | Sep 14, 2010 | A-343 | *V. campbellii* |
|  | *Madracis decactis* | Sep 14, 2010 | A-344 | *Vibrio* sp*.* |
|  | *Madracis decactis* | Sep 14, 2010 | A-345 | *V. campbellii* |
|  | *Madracis decactis* | Sep 14, 2010 | A-346 | *V. campbellii* |
|  | *Madracis decactis* | Sep 14, 2010 | A-347 | *V. campbellii* |
|  | *Madracis decactis* | Sep 14, 2010 | A-348 | *V. maritimus/V.variabilis group* |
|  | *Madracis decactis* | Sep 14, 2010 | A-349 | *V. campbellii* |
|  | *Madracis decactis* | Sep 14, 2010 | A-350 | *V. campbellii* |
|  | *Madracis decactis* | Sep 14, 2010 | A-351 | *V. harveyi* |
|  | *Madracis decactis* | Sep 14, 2010 | A-352 | *V. campbellii* |
|  | *Madracis decactis* | Sep 14, 2010 | A-353 | *Vibrio* sp*.* |
|  | *Madracis decactis* | Sep 14, 2010 | A-354 | *V. madracius* |
|  | *Madracis decactis* | Sep 14, 2010 | A-355 | *Vibrio* sp*.* |
|  | *Madracis decactis* | Sep 14, 2010 | A-356 | *V. harveyi* |
|  | *Madracis decactis* | Sep 14, 2010 | A-357 | *V. harveyi* |
|  | *Madracis decactis* | Sep 14, 2010 | A-358 | *V. harveyi* |
|  | *Madracis decactis* | Sep 14, 2010 | A-362 | *V. campbellii* |
|  | *Madracis decactis* | Sep 14, 2010 | A-363 | *V. chagasii* |
|  | *Madracis decactis* | Sep 14, 2010 | A-364 | *V. campbellii* |
|  | *Madracis decactis* | Sep 14, 2010 | A-365 | *V. campbellii* |
|  | *Madracis decactis* | Sep 14, 2010 | A-366 | *V. campbellii* |
|  | *Madracis decactis* | Sep 14, 2010 | A-367 | *V. maritimus* |
|  | *Madracis decactis* | Sep 14, 2010 | A-368 | *V. campbellii* |
|  | *Madracis decactis* | Sep 14, 2010 | A-369 | *V. campbellii* |
|  | *Madracis decactis* | Sep 14, 2010 | A-370 | *V. communis* |
|  | *Madracis decactis* | Sep 14, 2010 | A-371 | *V. communis* |
|  | *Madracis decactis* | Sep 14, 2010 | A-372 | *V. communis* |
|  | *Madracis decactis* | Sep 14, 2010 | A-374 | *V. campbellii* |
|  | *Madracis decactis* | Sep 14, 2010 | A-375 | *V. maritimus* |
|  | *Madracis decactis* | Sep 14, 2010 | A-376 | *V. communis* |
|  | *Madracis decactis* | Sep 14, 2010 | A-377 | *V. campbellii* |
|  | *Madracis decactis* | Sep 14, 2010 | A-378 | *Vibrio* sp*.* |
|  | *Madracis decactis* | Sep 14, 2010 | A-380 | *V. maritimus* |
|  | *Madracis decactis* | Sep 14, 2010 | A-381 | *V. harveyi* |
|  | *Madracis decactis* | Sep 14, 2010 | A-382 | *V. harveyi* |
|  | *Madracis decactis* | Sep 14, 2010 | A-383 | *Vibrio* sp*.* |
|  | *Madracis decactis* | Sep 14, 2010 | A-384 | *V. madracius* |
|  | *Madracis decactis* | Sep 14, 2010 | A-385 | *V. ponticus* |
|  | *Madracis decactis* | Sep 14, 2010 | A-386 | *V. ponticus* |
|  | *Madracis decactis* | Sep 14, 2010 | A-387 | *V. ponticus* |
|  | *Madracis decactis* | Sep 14, 2010 | A-389 | *Vibrio* sp. |
|  | *Madracis decactis* | Sep 14, 2010 | A-390 | *Vibrio* sp. |
|  | *Madracis decactis* | Sep 14, 2010 | A-391 | *V. campbellii* |
|  | *Madracis decactis* | Sep 14, 2010 | A-392 | *V. maritimus* |
|  | *Madracis decactis* | Sep 14, 2010 | A-393 | *V. chagasii* |
| **Saint Sebastian Channel _ São Paulo State** | *Mussismilia hispida* | March 2, 2005 | R-1 | *V. alginolyticus* |
|  | *Mussismilia hispida* | March 2, 2005 | R-2 | *V. parahaemolyticus* |
|  | *Mussismilia hispida* | March 2, 2005 | R-4 | *V. campbellii* |
|  | *Mussismilia hispida* | March 2, 2005 | R-6 | *V. chagasii* |
|  | *Mussismilia hispida* | March 2, 2005 | R-7 | *V. alginolyticus* |
|  | *Mussismilia hispida* | March 2, 2005 | R-9 | *V. alginolyticus* |
|  | *Mussismilia hispida* | March 2, 2005 | R-10 | *V. rotiferianus* |
|  | *Mussismilia hispida* | March 2, 2005 | R-11 | *V. communis* |
|  | *Palythoa caribaeorum* | March 2, 2005 | R-12 | *V. mediterranei* |
|  | *Palythoa caribaeorum* | March 2, 2005 | R-18 | *V. mediterranei* |
|  | *Mussismilia hispida* | March 2, 2005 | R-21 | *V. alginolyticus* |
|  | *Mussismilia hispida* | March 2, 2005 | R-22 | *V. diabolicus* |
|  | *Mussismilia hispida* | March 2, 2005 | R-26 | *V. chagasii* |
|  | *Palythoa caribaeorum* | March 2, 2005 | R-35 | *V. tubiashii* |
|  | *Palythoa caribaeorum* | March 2, 2005 | R-36 | *V. mediterranei* |
|  | *Palythoa caribaeorum* | March 2, 2005 | R-39 | *V. rotiferianus* |
|  | *Palythoa caribaeorum* | March 2, 2005 | R-40 | *V. harveyi* |
|  | *Palythoa caribaeorum* | March 2, 2005 | R-41 | *V. harveyi* |
|  | *Palythoa caribaeorum* | March 2, 2005 | R-42 | *V. rotiferianus* |
|  | *Mussismilia hispida* | March 2, 2005 | R-47 | *V. chagasii* |
|  | *Mussismilia hispida* | March 2, 2005 | R-48 | *V. chagasii* |
|  | *Mussismilia hispida* | March 2, 2005 | R-49 | *V. chagasii* |
|  | *Mussismilia hispida* | March 2, 2005 | R-50 | *V. harveyi* |
|  | *Mussismilia hispida* | March 2, 2005 | R-52 | *V. chagasii* |
|  | *Mussismilia hispida* | March 2, 2005 | R-54 | *V. communis* |
|  | *Mussismilia hispida* | March 2, 2005 | R-58 | *V. mediterranei* |
|  | *Palythoa variabilis* | March 2, 2005 | R-63 | *V. alginolyticus* |
|  | *Palythoa caribaeorum* | March 2, 2005 | R-77 | *V.maritimus group* |
|  | *Palythoa caribaeorum* | March 2, 2005 | R-78 | *V.variabilis* |
|  | *Palythoa variabilis* | March 2, 2005 | R-86 | *V. communis* |
|  | *Palythoa caribaeorum* | March 2, 2005 | R-91 | *V.maritimus* |
|  | *Mussismilia hispida* | March 2, 2005 | R-112 | *V. fortis* |
|  | *Mussismilia hispida* | March 2, 2005 | R-115 | *V. chagasii* |
|  | *Mussismilia hispida* | March 2, 2005 | R-123 | *V. communis* |
|  | *Mussismilia hispida* | March 2, 2005 | R-227 | *V. communis* |
|  | *Zoanthus solanderi* | March 2, 2005 | R-228 | *V. alginolyticus* |
|  | *Palythoa caribaeorum* | March 2, 2005 | R-229 | *V. tubiashii* |
|  | *Palythoa caribaeorum* | March 2, 2005 | R-230 | *V. harveyi* |
|  | *Zoanthus solanderi* | March 2, 2005 | R-232 | *V. alginolyticus* |
|  | *Mussismilia hispida* | March 2, 2005 | R-233 | *V. communis* |
|  | *Mussismilia hispida* | March 2, 2005 | R-234 | *V. alginolyticus* |
|  | *Zoanthus solanderi* | March 2, 2005 | R-235 | *V. alginolyticus* |
|  | *Mussismilia hispida* | March 2, 2005 | R-239 | *V. communis* |
|  | *Palythoa caribaeorum* | March 2, 2005 | R-240 | *Vibrio sp.* |
|  | *Palythoa caribaeorum* | March 2, 2005 | R-241 | *V. parahaemolyticus* |
|  | *Palythoa caribaeorum* | March 2, 2005 | R-242 | *V. harveyi* |
|  | *Palythoa caribaeorum* | March 2, 2005 | R-243 | *V. harveyi* |
|  | *Mussismilia hispida* | March 2, 2005 | R-246 | *V. harveyi* |
|  | *Mussismilia hispida* | March 2, 2005 | R-248 | *V. fortis* |
|  | *Palythoa caribaeorum* | March 2, 2005 | R-252 | *V. tubiashii* |
|  | *Mussismilia hispida* | March 2, 2005 | R-253 | *V. communis* |
|  | *Mussismilia hispida* | March 2, 2005 | R-254 | *V. fortis* |
|  | *Palythoa caribaeorum* | March 2, 2005 | R-255 | *V. communis* |
|  | *Palythoa caribaeorum* | March 2, 2005 | R-257 | *V. harveyi* |
|  | *Palythoa caribaeorum* | March 2, 2005 | R-259 | *V. harveyi* |
|  | *Palythoa caribaeorum* | March 2, 2005 | R-260 | *V. communis* |
|  | *Mussismilia hispida* | March 2, 2005 | R-262 | *V. alginolyticus* |
|  | *Mussismilia hispida* | March 2, 2005 | R-263 | *V. alginolyticus* |
|  | *Mussismilia hispida* | March 2, 2005 | R-264 | *V. communis* |
|  | *Palythoa caribaeorum* | March 2, 2005 | R-265 | *V. alginolyticus* |
|  | *Palythoa variabilis* | March 2, 2005 | R-280 | *V. communis* |
|  | *Zoanthus solanderi* | March 2, 2005 | R-283 | *V. alginolyticus* |
|  | *Mussismilia hispida* | March 2, 2005 | R-284 | *V. alginolyticus* |
|  | *Palythoa caribaeorum* | March 2, 2005 | R-285 | *V. harveyi* |
|  | *Palythoa caribaeorum* | March 2, 2005 | R-286 | *V. harveyi* |
|  | *Mussismilia hispida* | March 2, 2005 | R-287 | *V. alginolyticus* |
|  | *Mussismilia hispida* | March 2, 2005 | R-288 | *V. alginolyticus* |
|  | *Mussismilia hispida* | March 2, 2005 | R-289 | *V. alginolyticus* |
|  | *Mussismilia hispida* | March 2, 2005 | R-290 | *V. alginolyticus* |
|  | *Mussismilia hispida* | March 2, 2005 | R-291 | *V. alginolyticus* |
|  | *Mussismilia hispida* | March 2, 2005 | R-292 | *V. alginolyticus* |
|  | *Mussismilia hispida* | March 2, 2005 | R-293 | *V. alginolyticus* |
|  | *Zoanthus solanderi* | March 2, 2005 | R-294 | *V. alginolyticus* |
|  | *Palythoa variabilis* | March 2, 2005 | R-295 | *V. alginolyticus* |
|  | *Palythoa caribaeorum* | March 2, 2005 | R-296 | *V. alginolyticus* |
|  | *Mussismilia hispida* | March 2, 2005 | R-297 | *V. alginolyticus* |
|  | *Mussismilia hispida* | March 2, 2005 | R-298 | *V. alginolyticus* |
|  | *Mussismilia hispida* | March 2, 2005 | R-299 | *V. alginolyticus* |
|  | *Mussismilia hispida* | March 2, 2005 | R-300 | *V. alginolyticus* |
|  | *Palythoa variabilis* | March 2, 2005 | R-301 | *V. alginolyticus* |
|  | *Mussismilia hispida* | March 2, 2005 | R-302 | *V. alginolyticus* |
|  | *Mussismilia hispida* | March 2, 2005 | R-303 | *V. alginolyticus* |
|  | *Mussismilia hispida* | March 2, 2005 | R-304 | *V. alginolyticus* |
|  | *Palythoa caribaeorum* | March 2, 2005 | R-305 | *V. harveyi* |
|  | *Mussismilia hispida* | March 2, 2005 | R-306 | *V. alginolyticus* |
|  | *Palythoa caribaeorum* | March 2, 2005 | R-307 | *V. harveyi* |
|  | *Zoanthus solanderi* | March 2, 2005 | R-308 | *V. alginolyticus* |
|  | *Mussismilia hispida* | March 2, 2005 | R-309 | *V. alginolyticus* |
|  | *Mussismilia hispida* | March 2, 2005 | R-310 | *V. alginolyticus* |
|  | *Palythoa caribaeorum* | March 2, 2005 | R-311 | *V. harveyi* |
|  | *Mussismilia hispida* | March 2, 2005 | R-312 | *V. alginolyticus* |
|  | *Palythoa variabilis* | March 2, 2005 | R-313 | *V. alginolyticus* |
|  | *Mussismilia hispida* | March 2, 2005 | R-314 | *V. alginolyticus* |
|  | *Mussismilia hispida* | March 2, 2005 | R-315 | *V. alginolyticus* |
|  | *Palythoa caribaeorum* | March 2, 2005 | R-316 | *V. alginolyticus* |
|  | *Palythoa caribaeorum* | March 2, 2005 | R-317 | *V. alginolyticus* |
|  | *Mussismilia hispida* | March 2, 2005 | R-318 | *V. alginolyticus* |
|  | *Palythoa caribaeorum* | March 2, 2005 | R-319 | *V. alginolyticus* |
|  | *Palythoa caribaeorum* | March 2, 2005 | R-320 | *V. alginolyticus* |
|  | *Mussismilia hispida* | March 2, 2005 | R-321 | *V. alginolyticus* |
|  | *Mussismilia hispida* | March 2, 2005 | R-322 | *V. alginolyticus* |
|  | *Mussismilia hispida* | March 2, 2005 | R-323 | *V. alginolyticus* |
|  | *Mussismilia hispida* | March 2, 2005 | R-324 | *V. alginolyticus* |
|  | *Palythoa caribaeorum* | March 2, 2005 | R-325 | *V. alginolyticus* |
|  | *Mussismilia hispida* | March 2, 2005 | R-326 | *V. alginolyticus* |
|  | *Palythoa caribaeorum* | March 2, 2005 | R-327 | *V. harveyi* |
|  | *Palythoa caribaeorum* | March 2, 2005 | R-328 | *V. harveyi* |
|  | *Zoanthus solanderi* | March 2, 2005 | R-329 | *V. alginolyticus* |
|  | *Palythoa caribaeorum* | March 2, 2005 | R-330 | *V. harveyi* |
|  | *Palythoa variabilis* | March 2, 2005 | R-331 | *V. alginolyticus* |
|  | *Mussismilia hispida* | January 2, 2006 | R-600 | *V. communis* |
|  | *Mussismilia hispida* | January 2, 2006 | R-604 | *V. campbellii* |
|  | *Mussismilia hispida* | January 2, 2006 | R-608 | *V. campbellii* |
|  | *Mussismilia hispida* | January 2, 2006 | R-609 | *V. campbellii* |
|  | *Mussismilia hispida* | January 2, 2006 | R-610 | *V. communis* |
|  | *Mussismilia hispida* | January 2, 2006 | R-612 | *V. campbelii* |
|  | *Mussismilia hispida* | January 2, 2006 | R-613 | *V. communis* |
|  | *Mussismilia hispida* | January 2, 2006 | R-614 | *V. sinaloensis* |
|  | *Mussismilia hispida* | January 2, 2006 | R-616 | *V. maritimus* |
|  | *Mussismilia hispida* | January 2, 2006 | R-617 | *V. communis* |
|  | *Mussismilia hispida* | January 2, 2006 | R-618 | *V. communis* |
|  | *Mussismilia hispida* | January 2, 2006 | R-619 | *V. maritimus* |
|  | *Mussismilia hispida* | January 2, 2006 | R-620 | *V. communis* |
|  | *Palythoa caribaeorum* | January 2, 2006 | R-621 | *V. harveyi* |
|  | *Palythoa caribaeorum* | January 2, 2006 | R-622 | *V. harveyi* |
|  | *Mussismilia hispida* | January 2, 2006 | R-624 | *V. alginolyticus* |
|  | *Mussismilia hispida* | January 2, 2006 | R-627 | *V. sinaloensis* |
|  | *Mussismilia hispida* | January 2, 2006 | R-628 | *V. communis* |
|  | *Mussismilia hispida* | January 2, 2006 | R-629 | *V. communis* |
|  | *Mussismilia hispida* | January 2, 2006 | R-630 | *V. communis* |
|  | *Mussismilia hispida* | January 2, 2006 | R-631 | *V. communis* |
|  | *Mussismilia hispida* | January 2, 2006 | R-632 | *V. communis* |
|  | *Mussismilia hispida* | January 2, 2006 | R-634 | *V. communis* |
|  | *Mussismilia hispida* | January 2, 2006 | R-635 | *V. alginolyticus* |
|  | *Mussismilia hispida* | January 2, 2006 | R-637 | *V. campbelii* |
|  | *Mussismilia hispida* | January 2, 2006 | R-638 | *V. mediterranei* |
|  | *Mussismilia hispida* | January 2, 2006 | R-639 | *V. mediterranei* |
|  | *Mussismilia hispida* | January 2, 2006 | R-641 | *V. sinaloensis* |
|  | *Mussismilia hispida* | January 2, 2006 | R-642 | *V. communis* |
|  | *Mussismilia hispida* | January 2, 2006 | R-643 | *V. pelagius* |
|  | *Mussismilia hispida* | January 2, 2006 | R-645 | *V. campbellii* |
|  | *Mussismilia hispida* | January 2, 2006 | R-647 | *V. communis* |
|  | *Mussismilia hispida* | January 2, 2006 | R-648 | *V. mediterranei* |
|  | *Mussismilia hispida* | January 2, 2006 | R-649 | *V. campbellii* |
|  | *Mussismilia hispida* | January 2, 2006 | R-650 | *V. communis* |
|  | *Mussismilia hispida* | January 2, 2006 | R-651 | *V. communis* |
|  | *Mussismilia hispida* | January 2, 2006 | R-654 | *V. sinaloensis* |
|  | *Mussismilia hispida* | January 2, 2006 | R-656 | *V. campbellii* |
|  | *Mussismilia hispida* | January 2, 2006 | R-659 | *V. tubiashii* |
|  | *Mussismilia hispida* | January 2, 2006 | R-660 | *V. mediterranei* |
|  | *Mussismilia hispida* | January 2, 2006 | R-661 | *V. mediterranei* |
|  | *Palythoa caribaeorum* | January 2, 2006 | R-662 | *V. harveyi* |
|  | *Mussismilia hispida* | January 2, 2006 | R-663 | *V. mediterranei* |
|  | *Mussismilia hispida* | January 2, 2006 | R-664 | *V. communis* |
|  | *Mussismilia hispida* | January 2, 2006 | R-665 | *V. alginolyticus* |
|  | *Mussismilia hispida* | January 2, 2006 | R-666 | *V. alginolyticus* |
|  | *Mussismilia hispida* | January 2, 2006 | R-667 | *V. communis* |
|  | *Mussismilia hispida* | January 2, 2006 | R-668 | *V. communis* |
|  | *Mussismilia hispida* | January 2, 2006 | R-669 | *V. communis* |
|  | *Mussismilia hispida* | January 2, 2006 | R-670 | *V. chagasii* |
|  | *Mussismilia hispida* | January 2, 2006 | R-671 | *V. communis* |
|  | *Mussismilia hispida* | January 2, 2006 | R-672 | *V. communis* |
|  | *Mussismilia hispida* | January 2, 2006 | R-673 | *V. communis* |
|  | *Mussismilia hispida* | January 2, 2006 | R-674 | *V. tubiashii* |
|  | *Palythoa caribaeorum* | January 2, 2006 | R-676 | *V. tubiashii* |
|  | *Palythoa caribaeorum* | January 2, 2006 | R-677 | *V. tubiashii* |
|  | *Palythoa caribaeorum* | January 2, 2006 | R-678 | *V. tubiashii* |
|  | *Palythoa caribaeorum* | January 2, 2006 | R-679 | *V. communis* |
|  | *Mussismilia hispida* | January 2, 2006 | R-680 | *V. communis* |
|  | *Mussismilia hispida* | January 2, 2006 | R-681 | *V. communis* |
|  | *Mussismilia hispida* | January 2, 2006 | R-682 | *V. rotiferianus* |
|  | *Mussismilia hispida* | January 2, 2006 | R-683 | *V. chagasii* |
|  | *Mussismilia hispida* | January 2, 2006 | R-684 | *V. campbelii* |
|  | *Mussismilia hispida* | January 2, 2006 | R-685 | *V. mediterranei* |
|  | *Mussismilia hispida* | January 2, 2006 | R-686 | *V. mediterranei* |
|  | *Mussismilia hispida* | January 2, 2006 | R-687 | *V. communis* |
|  | *Palythoa caribaeorum* | January 2, 2006 | R-688 | *V. harveyi* |
|  | *Mussismilia hispida* | January 2, 2006 | R-690 | *V. harveyi* |
|  | *Palythoa caribaeorum* | January 2, 2006 | R-691 | *V. communis* |
|  | *Palythoa caribaeorum* | January 2, 2006 | R-692 | *V. harveyi* |
|  | *Palythoa caribaeorum* | January 2, 2006 | R-693 | *V. harveyi* |
|  | *Palythoa caribaeorum* | January 2, 2006 | R-694 | *V. harveyi* |
|  | *Mussismilia hispida* | January 2, 2006 | R-695 | *V. communis* |
|  | *Mussismilia hispida* | January 2, 2006 | R-696 | *V. communis* |
|  | *Mussismilia hispida* | January 2, 2006 | R-697 | *V. communis* |
|  | *Mussismilia hispida* | January 2, 2006 | R-699 | *V. communis* |
|  | *Mussismilia hispida* | January 2, 2006 | R-700 | *V. communis* |
|  | *Mussismilia hispida* | January 2, 2006 | R-701 | *V. sinaloensis* |
|  | *Mussismilia hispida* | January 2, 2006 | R-705 | *V. communis* |
|  | *Mussismilia hispida* | January 2, 2006 | R-707 | *V. ponticus* |
|  | *Palythoa caribaeorum* | January 2, 2006 | R-708 | *V. alginolyticus* |
|  | *Mussismilia hispida* | January 2, 2006 | R-709 | *V. communis* |
|  | *Palythoa caribaeorum* | January 2, 2006 | R-710 | *V. campbellii* |
|  | *Palythoa caribaeorum* | January 2, 2006 | R-711 | *V. tubiashii* |
|  | *Palythoa caribaeorum* | January 2, 2006 | R-712 | *V. campbellii* |
|  | *Mussismilia hispida* | January 2, 2006 | R-713 | *V. mediterranei* |
|  | *Mussismilia hispida* | January 2, 2006 | R-715 | *V. cincinnatiensis* |
|  | *Mussismilia hispida* | January 2, 2006 | R-716 | *V. tubiashii* |
|  | *Palythoa caribaeorum* | January 2, 2006 | R-718 | *V. harveyi* |
|  | *Mussismilia hispida* | January 2, 2006 | R-719 | *V. mediterranei* |
|  | *Mussismilia hispida* | January 2, 2006 | R-720 | *V. mediterranei* |
|  | *Mussismilia hispida* | January 2, 2006 | R-722 | *V. tubiashii* |
|  | *Mussismilia hispida* | January 2, 2006 | R-723 | *V. tubiashii* |
|  | *Mussismilia hispida* | January 2, 2006 | R-725 | *V. communis* |
|  | *Mussismilia hispida* | January 2, 2006 | R-726 | *V. communis* |
|  | *Mussismilia hispida* | January 2, 2006 | R-729 | *V. communis* |
|  | *Mussismilia hispida* | January 2, 2006 | R-731 | *V. tubiashii* |
|  | *Mussismilia hispida* | January 2, 2006 | R-732 | *V. sinaloensis* |
|  | *Mussismilia hispida* | January 2, 2006 | R-775 | *V. communis* |
