# Supplementary material for: Diversity and ecological structure of vibrios in benthic and pelagic habitats along a latitudinal gradient in the Southwest Atlantic Ocean
Source: PeerJ. 2015 Feb 10;3:e741. doi: 10.7717/peerj.741 (PMC4327252; doi:10.7717/peerj.741)
Supplement: Table S2 [file peerj-03-741-s008.docx]

| **Habitats spectrum** | **Vibrio species associated with local and source isolation** |
| --- | --- |
| habitat 1 | AW_P106A/V. maritimus |
| habitat 1 | AW_P102B/V. maritimus |
| habitat 1 | AW_P111A/V. maritimus |
| habitat 1 | AW_P124A/V. maritimus |
| habitat 1 | AW_P122A/V. maritimus |
| habitat 1 | AW_P125A/V. maritimus |
| habitat 1 | AW_P121C/V. maritimus |
| habitat 1 | AW_P125B/V. maritimus |
| habitat 1 | AW_P102A/V. maritimus |
| habitat 1 | SA_R77/V. maritimus group |
| habitat 1 | AW_P21C/V. maritimus group |
| habitat 1 | AW_P21D/V. maritimus group |
| habitat 1 | AW_P21G/V. maritimus group |
| habitat 1 | AW_P21E/V. maritimus group |
| habitat 1 | AW_P21F/V. maritimus group |
| habitat 1 | AW_P21B/V. maritimus group |
| habitat 1 | AW_P21A/V. maritimus group |
| habitat 1 | AW_P37A/V.diabolicus |
| habitat 1 | AW_P41B/V.diabolicus |
| habitat 1 | AW_P37C/V.diabolicus |
| habitat 1 | AW_P40E/V.diabolicus |
| habitat 1 | AW_P38A/V.diabolicus |
| habitat 1 | AW_P41D/V.diabolicus |
| habitat 1 | AW_P40D/V.diabolicus |
| habitat 1 | AW_P37B/V.diabolicus |
| habitat 1 | AW_P38B/V.diabolicus |
| habitat 1 | AW_P38C/V.diabolicus |
| habitat 1 | AW_P40C/V.diabolicus |
| habitat 1 | AW_P118A/V.dibolicus |
| habitat 1 | AW_P118B/V.dibolicus |
| habitat 1 | FP_4D2/V.diabolicus |
| habitat 1 | AW_P40B/V.diabolicus |
| habitat 1 | AW_P23D/V.campbellii |
| habitat 1 | AW_P23C/V.campbellii |
| habitat 1 | AW_P48B/V.campbellii |
| habitat 1 | FP_4SA6/V.campbellii |
| habitat 1 | RH_42A/V.campbellii |
| habitat 1 | AW_P44A/V.campbellii |
| habitat 1 | AW_P45A/V.campbellii |
| habitat 1 | AW_P104A/V. communis |
| habitat 1 | SH_R239/V. communis |
| habitat 1 | MH_50B/V. communis |
| habitat 1 | AW_P105A/V. communis |
| habitat 1 | SV_R280/V. communis |
| habitat 1 | AW_P103A/V. communis |
| habitat 1 | SH_R264/V. communis |
| habitat 1 | AW_P109A/V. communis |
| habitat 1 | AW_P108A/V. communis |
| habitat 1 | AW_P107A/V. communis |
| habitat 1 | AW_P112A/V. communis |
| habitat 1 | AW_P110A/V. communis |
| habitat 1 | AW_P48A/V. communis |
| habitat 1 | AW_P20C/V. hepatarius |
| habitat 1 | AW_P20G/V. hepatarius |
| habitat 1 | AW_P20A/V. hepatarius |
| habitat 1 | AW_P20F/V. hepatarius |
| habitat 1 | AW_P20E/V. hepatarius |
| habitat 1 | AW_P20H/V. hepatarius |
| habitat 1 | AW_P20B/V. hepatarius |
| habitat 1 | AW_P115B/V. pelagius |
| habitat 1 | AW_P115F/V. pelagius |
| habitat 1 | AW_P115E/V. pelagius |
| habitat 1 | AW_P115C/V. pelagius |
| habitat 1 | AW_P115H/V. pelagius |
| habitat 1 | AW_P115G/V. pelagius |
| habitat 1 | AW_P115J/V. pelagius |
| habitat 1 | AW_P115A/V. pelagius |
| habitat 1 | AW_P115D/V. pelagius |
| habitat 1 | AW_P22B/V. pelagius |
| habitat 1 | MH_28A2/V. pelagius |
| habitat 1 | AW_P47A/V. chagasii |
| habitat 1 | IB_PA10/V. chagasii |
| habitat 2 | PD_A320/V. maritimus/V.variabilis group |
| habitat 2 | PD_A322/V. maritimus/V.variabilis group |
| habitat 2 | PD_A348/V. maritimus/V.variabilis group |
| habitat 2 | PD_A297/V. maritimus/V.variabilis group |
| habitat 2 | PD_A338/V. maritimus/V.variabilis group |
| habitat 2 | PD_A107/V. maritimus |
| habitat 2 | PD_A106/V. maritimus |
| habitat 2 | PD_A340/V. maritimus |
| habitat 2 | PD_A367/V. maritimus |
| habitat 2 | PD_A380/V. maritimus |
| habitat 2 | PD_A321/V. maritimus/V.variabilis group |
| habitat 2 | PD_A392/V. maritimus |
| habitat 2 | PD_A327/V. maritimus |
| habitat 2 | PD_A115/V. maritimus |
| habitat 2 | PD_A375/V. maritimus |
| habitat 2 | PD_A344/V. sp |
| habitat 2 | PD_A390/V. sp |
| habitat 2 | PD_A353/V. sp |
| habitat 2 | PD_A305/V. sp |
| habitat 2 | PD_A307/V. sp |
| habitat 2 | PD_A383/V. sp |
| habitat 2 | PD_A306/V. sp |
| habitat 2 | PD_A355/V. sp |
| habitat 2 | PD_A302/V. sp |
| habitat 2 | PD_A312/V. sp |
| habitat 2 | PD_A303/V. sp |
| habitat 2 | PD_A96/V. sp |
| habitat 2 | PD_A103/V. sp |
| habitat 2 | PD_A110/V. sp |
| habitat 2 | PD_A378/V. sp |
| habitat 2 | PD_A389/V. sp |
| habitat 2 | PD_A314/V. sp |
| habitat 2 | PC_A295/V. sp |
| habitat 2 | PD_A384/V.madracius |
| habitat 2 | PD_A328/V.madracius |
| habitat 2 | PD_A354/V.madracius |
| habitat 2 | PD_A326/Vibrio sp. |
| habitat 2 | PD_A386/V. ponticus |
| habitat 2 | PD_A387/V. ponticus |
| habitat 2 | PD_A385/V. ponticus |
| habitat 2 | PD_A349/V.campbellii |
| habitat 2 | PD_A377/V.campbellii |
| habitat 2 | AW_P23A/V.campbellii |
| habitat 2 | PD_A342/V.campbellii |
| habitat 2 | FP_2SA4/V.campbellii |
| habitat 2 | PD_A318/V.campbellii |
| habitat 2 | PD_A101/V.campbellii |
| habitat 2 | PD_A100/V.campbellii |
| habitat 2 | PD_A109/V.campbellii |
| habitat 2 | PD_A341/V.campbellii |
| habitat 2 | PD_A369/V.campbellii |
| habitat 2 | PD_A368/V.campbellii |
| habitat 2 | PD_A97/V.campbellii |
| habitat 2 | AW_P23B/V.campbellii |
| habitat 2 | PD_A347/V.campbellii |
| habitat 2 | PD_A104/V.campbellii |
| habitat 2 | PD_A105/V.campbellii |
| habitat 2 | AW_P22A/V.campbellii |
| habitat 2 | AW_P22C/V.campbellii |
| habitat 2 | PD_A346/V.campbellii |
| habitat 2 | PD_A111/V.campbellii |
| habitat 2 | PD_A98/V.campbellii |
| habitat 2 | PD_A102/V.campbellii |
| habitat 2 | PD_A311/V.campbellii |
| habitat 2 | PD_A362/V.campbellii |
| habitat 2 | PD_A350/V.campbellii |
| habitat 2 | PD_A296/V.campbellii |
| habitat 2 | PD_A343/V.campbellii |
| habitat 2 | PD_A352/V.campbellii |
| habitat 2 | PD_A95/V.campbellii |
| habitat 2 | PD_A108/V.campbellii |
| habitat 2 | PD_A364/V.campbellii |
| habitat 2 | PD_A366/V.campbellii |
| habitat 2 | PD_A365/V.campbellii |
| habitat 2 | SH_R609/V.campbellii |
| habitat 2 | SH_R612/V.campbellii |
| habitat 2 | PD_A334/V.campbellii |
| habitat 2 | PD_A330/V.campbellii |
| habitat 2 | PD_A374/V.campbellii |
| habitat 2 | PD_A391/V.campbellii |
| habitat 2 | PD_A333/V.campbellii |
| habitat 2 | PD_A345/V.campbellii |
| habitat 2 | PD_A370/V. communis |
| habitat 2 | PD_A299/V. communis |
| habitat 2 | PD_A300/V. communis |
| habitat 2 | PD_A337/V. communis |
| habitat 2 | PD_A298/V. communis |
| habitat 2 | PD_A291/V. communis |
| habitat 2 | PD_A293/V. communis |
| habitat 2 | PD_A292/V. communis |
| habitat 2 | PD_A290/V. communis |
| habitat 2 | PD_A335/V. communis |
| habitat 2 | PD_A324/V. pelagius |
| habitat 2 | PD_A323/V. pelagius |
| habitat 2 | PD_A325/V. pelagius |
| habitat 2 | PD_A329/V. pelagius |
| habitat 2 | PD_A363/V. chagasii |
| habitat 2 | PD_A393/V. chagasii |
| habitat 3 | RB_74G/V. harveyi |
| habitat 3 | SA_R328/V. harveyi |
| habitat 3 | BR_G13/V. harveyi |
| habitat 3 | BR_G46/V. harveyi |
| habitat 3 | SA_R327/V. harveyi |
| habitat 3 | PS_A57/V. harveyi |
| habitat 3 | SA_R242/V. harveyi |
| habitat 3 | SH_R246/V. harveyi |
| habitat 3 | RB_74I/V. harveyi |
| habitat 3 | BR_G32/V. harveyi |
| habitat 3 | RB_38C/V. campbellii |
| habitat 3 | RB_38B/V. communis |
| habitat 3 | RB_74C/V. communis |
| habitat 3 | BE_1/V. communis |
| habitat 3 | RB_74B/V. communis |
| habitat 3 | RB_74D/V. communis |
| habitat 3 | RB_74O/V. communis |
| habitat 3 | RB_74P/V. communis |
| habitat 3 | RB_74N/V. communis |
| habitat 3 | RB_74Q/V. communis |
| habitat 3 | RB_74M/V. communis |
| habitat 3 | BE_20/V. communis |
| habitat 3 | SH_R617/V. communis |
| habitat 3 | BE_3/V. coralliilyticus |
| habitat 3 | RB_38A/V. coralliilyticus |
| habitat 3 | RH_42B/V. coralliilyticus |
| habitat 3 | FP_3DA5/V. coralliilyticus |
| habitat 3 | MB_45B/V. coralliilyticus |
| habitat 3 | FP_4SA5/V. coralliilyticus |
| habitat 3 | MB_45D/V. coralliilyticus |
| habitat 3 | RB_43B/V. coralliilyticus |
| habitat 3 | RB_43P/V. coralliilyticus |
| habitat 3 | RB_43Q/V. coralliilyticus |
| habitat 3 | RB_43AC/V. coralliilyticus |
| habitat 3 | RB_43O/V. coralliilyticus |
| habitat 3 | BR_G19/V. coralliilyticus |
| habitat 3 | RB_43C/V. coralliilyticus |
| habitat 3 | MB_45C/V. coralliilyticus |
| habitat 3 | RB_43Z/V. tubiashii |
| habitat 3 | RB_43I/V. tubiashii |
| habitat 3 | RB_74K/V. tubiashii |
| habitat 3 | RB_74J/V. tubiashii |
| habitat 3 | RB_43K/V. tubiashii |
| habitat 3 | RB_43/V. sinaloensis |
| habitat 3 | RB_43Y/V. sinaloensis |
| habitat 3 | RB_43U/V. sinaloensis |
| habitat 3 | RB_43AD/V. sinaloensis |
| habitat 3 | RB_43X/V. sinaloensis |
| habitat 3 | RB_43AH/V. sinaloensis |
| habitat 3 | RB_43AM/V. sinaloensis |
| habitat 4 | SA_R91/V. maritimus |
| habitat 4 | SH_R616/V. maritimus |
| habitat 4 | SH_R619/V. maritimus |
| habitat 4 | SA_R36/V. meditarranei |
| habitat 4 | SH_R648/V. meditarranei |
| habitat 4 | SH_R18/V. meditarranei |
| habitat 4 | SH_R58/V. meditarranei |
| habitat 4 | SA_R325/V. alginolyticus |
| habitat 4 | SH_R7/V. alginolyticus |
| habitat 4 | SH_R326/V. alginolyticus |
| habitat 4 | SH_R322/V. alginolyticus |
| habitat 4 | SH_R300/V. alginolyticus |
| habitat 4 | FP_2SA1/V. alginolyticus |
| habitat 4 | FP_1SA1/V. alginolyticus |
| habitat 4 | MH_27C/V. alginolyticus |
| habitat 4 | RB_40E/V. alginolyticusi |
| habitat 4 | FP_4SA7/V. alginolyticus |
| habitat 4 | SH_R635/V. alginolyticus |
| habitat 4 | MH_27A/V. alginolyticus |
| habitat 4 | SA_R296/V. alginolyticus |
| habitat 4 | SA_R265/V. alginolyticus |
| habitat 4 | SA_R319/V. alginolyticus |
| habitat 4 | FP_1D3/V. alginolyticus |
| habitat 4 | FP_3SA4/V. alginolyticus |
| habitat 4 | RB_40B/V. alginolyticus |
| habitat 4 | FP_3SA5/V. alginolyticus |
| habitat 4 | SH_R309/V. alginolyticus |
| habitat 4 | SH_R292/V. alginolyticus |
| habitat 4 | MH_27E/V. alginolyticus |
| habitat 4 | SZ_R329/V. alginolyticus |
| habitat 4 | SZ_R294/V. alginolyticus |
| habitat 4 | FP_1S4/V. alginolyticus |
| habitat 4 | SH_R288/V. alginolyticus |
| habitat 4 | SH_R304/V. alginolyticus |
| habitat 4 | SH_R666/V. alginolyticus |
| habitat 4 | FP_4S4/V. alginolyticus |
| habitat 4 | FP_1SA2/V. alginolyticus |
| habitat 4 | SZ_R235/V. alginolyticus |
| habitat 4 | SH_R263/V. alginolyticus |
| habitat 4 | SH_R299/V. alginolyticus |
| habitat 4 | SV_R301/V. alginolyticus |
| habitat 4 | SH_R1/V. alginolyticus |
| habitat 4 | SH_R262/V. alginolyticus |
| habitat 4 | SZ_R228/V. alginolyticus |
| habitat 4 | SZ_R232/V. alginolyticus |
| habitat 4 | SH_R321/V. alginolyticus |
| habitat 4 | SH_R302/V. alginolyticus |
| habitat 4 | SH_R290/V. alginolyticus |
| habitat 4 | SA_R708/V. alginolyticus |
| habitat 4 | SZ_R283/V. alginolyticus |
| habitat 4 | SH_R293/V. alginolyticus |
| habitat 4 | SZ_R308/V. alginolyticus |
| habitat 4 | SH_R289/V. alginolyticus |
| habitat 4 | SH_R291/V. alginolyticus |
| habitat 4 | SH_R234/V. alginolyticus |
| habitat 4 | SH_R324/V. alginolyticus |
| habitat 4 | SH_R323/V. alginolyticus |
| habitat 4 | SA_R317/V. alginolyticus |
| habitat 4 | SA_R316/V. alginolyticus |
| habitat 4 | SH_R314/V. alginolyticus |
| habitat 4 | SH_R315/V. alginolyticus |
| habitat 4 | SH_R284/V. alginolyticus |
| habitat 4 | SH_R312/V. alginolyticus |
| habitat 4 | SH_R310/V. alginolyticus |
| habitat 4 | SH_R306/V. alginolyticus |
| habitat 4 | SH_R21/V. alginolyticus |
| habitat 4 | SH_R9/V. alginolyticus |
| habitat 4 | SH_R303/V. alginolyticus |
| habitat 4 | SH_R665/V. alginolyticus |
| habitat 4 | SH_R624/V. alginolyticus |
| habitat 4 | SH_R318/V. alginolyticus |
| habitat 4 | SA_R712/V.campbellii |
| habitat 4 | SH_R4/V.campbellii |
| habitat 4 | SH_R684/V. campbellii |
| habitat 4 | SH_R645/V.campbellii |
| habitat 4 | SH_R649/V.campbellii |
| habitat 4 | SH_R608/V. campbellii |
| habitat 4 | SH_R637/V.campbellii |
| habitat 4 | SH_R604/V.campbellii |
| habitat 4 | SH_R656/V. campbellii |
| habitat 4 | SA_R710/V.campbellii |
| habitat 4 | SH_R642/V. communis |
| habitat 4 | SH_R618/V. communis |
| habitat 4 | SH_R687/V. communis |
| habitat 4 | SH_R671/V. communis |
| habitat 4 | SH_R663/V. meditarranei |
| habitat 4 | SH_R672/V. communis |
| habitat 4 | SH_R650/V. communis |
| habitat 4 | SH_R631/V. communis |
| habitat 4 | FP_2SA7/V. communis |
| habitat 4 | FP_2SA6/V. communis |
| habitat 4 | SH_R669/V. communis |
| habitat 4 | SH_R709/V. communis |
| habitat 4 | SH_R673/V. communis |
| habitat 4 | SH_R679/V. communis |
| habitat 4 | SH_R647/V. communis |
| habitat 4 | BE_30/V. communis |
| habitat 4 | SH_R630/V. communis |
| habitat 4 | BR_G38/V. communis |
| habitat 4 | SA_R691/V. communis |
| habitat 4 | SH_R253/V. communis |
| habitat 4 | BE_27/V. communis |
| habitat 4 | SH_R700/V. communis |
| habitat 4 | SH_R681/V. communis |
| habitat 4 | SH_R233/V. communis |
| habitat 4 | SH_R668/V. communis |
| habitat 4 | SH_R628/V. communis |
| habitat 4 | SA_R255/V. communis |
| habitat 4 | SH_R651/V. communis |
| habitat 4 | SH_R695/V. communis |
| habitat 4 | SH_R600/V. communis |
| habitat 4 | SH_R620/V. communis |
| habitat 4 | SH_R722/V. tubiashii |
| habitat 4 | SH_R731/V. tubiashii |
| habitat 4 | SH_R723/V. tubiashii |
| habitat 4 | SA_R711/V. tubiashii |
| habitat 4 | SH_R716/V. tubiashii |
| habitat 4 | SH_R659/V. tubiashii |
| habitat 4 | SH_R674/V. tubiashii |
| habitat 4 | SH_R627/V. sinaloensis |
| habitat 4 | SH_R654/V. sinaloensis |
| habitat 4 | SH_R732/V. sinaloensis |
| habitat 4 | SH_R614/V. sinaloensis |
| habitat 4 | SH_R701/V. sinaloensis |
| habitat 4 | SH_R715/V. cincinnatiensis |
| habitat 4 | SH_R641/V. sinaloensis |
| habitat 4 | SH_R112/V. fortis |
| habitat 4 | SH_R254/V. fortis |
| habitat 4 | SH_R248/V. fortis |
| habitat 4 | FP_1DA2/V. pelagius |
| habitat 4 | SH_R643/V. pelagius |
| habitat 4 | SH_R6/V. chagasii |
| habitat 4 | SH_R52/V. chagasii |
| habitat 4 | SH_R26//V. chagasii |
| habitat 4 | SH_R683/V. chagasii |
| habitat 4 | SH_R670/V. chagasii |
| habitat 4 | SH_R48/V. chagasii |
| habitat 4 | SH_R47/V. chagasii |
| habitat 4 | SH_R49/V. chagasii |
| habitat 4 | FP_1DA1/V. chagasii |
| habitat 4 | SH_R115/V. chagasii |
| habitat 5 | SV_R63/V. alginolyticus |
| habitat 5 | SA_R320/V. alginolyticus |
| habitat 5 | SH_R22/V. diabolicus |
| habitat 5 | FP_1D1/V. diabolicus |
| habitat 5 | SV_R331/V. alginolyticus |
| habitat 5 | SV_R295/V. alginolyticus |
| habitat 5 | SH_R298/V. alginolyticus |
| habitat 5 | SV_R313/V. alginolyticus |
| habitat 5 | SH_R297/V. alginolyticus |
| habitat 5 | MH_27B/V. alginolyticus |
| habitat 5 | SH_R287/V. alginolyticus |
| habitat 5 | MH_27D/V. alginolyticus |
| habitat 5 | RB_43R/V. communis |
| habitat 5 | FP_3D3/V. communis |
| habitat 5 | RB_43G/V. communis |
| habitat 5 | RB_43AF/V. communis |
| habitat 5 | MH_35B/V. communis |
| habitat 5 | RB_43S/V. communis |
| habitat 5 | SH_R705/V. communis |
| habitat 5 | SH_R775/V. communis |
| habitat 5 | RB_43T/V. communis |
| habitat 5 | RB_43H/V. communis |
| habitat 5 | RB_43AG/V. communis |
| habitat 5 | MH_35A/V. communis |
| habitat 5 | RB_43F/V. communis |
| habitat 5 | SH_R726/V. communis |
| habitat 5 | SH_R725/V. communis |
| habitat 5 | SH_R699/V. communis |
| habitat 5 | PD_A336/V. communis |
| habitat 5 | SH_R629/V. communis |
| habitat 5 | SH_R610/V. communis |
| habitat 5 | SH_R632/V. communis |
| habitat 5 | SH_R634/V. communis |
| habitat 5 | FP_3D1/V. communis |
| habitat 5 | MH_50F/V. communis |
| habitat 5 | FP_4SA3/V. communis |
| habitat 5 | MH_50D/V. communis |
| habitat 5 | MH_50A/V. communis |
| habitat 5 | MH_50G/V. communis |
| habitat 5 | MH_50C/V. communis |
| habitat 5 | RB_43AA/V. communis |
| habitat 5 | RB_43M/V. communis |
| habitat 5 | SH_R54/V. communis |
| habitat 5 | FP_4SA2/V. communis |
| habitat 5 | BR_G9/V. communis |
| habitat 5 | BR_G79/V. communis |
| habitat 5 | RB_43L/V. communis |
| habitat 5 | MH_12K/V. communis |
| habitat 5 | SH_R667/V. communis |
| habitat 5 | RB_43AB/V. communis |
| habitat 5 | RB_43N/V. communis |
| habitat 5 | FP_1DA3/V. communis |
| habitat 5 | RB_43E/V. communis |
| habitat 5 | BR_G57/V. communis |
| habitat 6 | BR_G69/V. sp |
| habitat 6 | SH_R707/V. ponticus |
| habitat 6 | AW_P119A/V. alfacsensis |
| habitat 6 | BR_G74/V. sp |
| habitat 6 | BR_G24/V. harveyi |
| habitat 6 | PD_A304/V. harveyi |
| habitat 6 | BE_29/V. harveyi |
| habitat 6 | BR_G26/V. harveyi |
| habitat 6 | PD_A357/V. harveyi |
| habitat 6 | BR_G21/V. harveyi |
| habitat 6 | MH_50E/V. harveyi |
| habitat 6 | BE_24/V. harveyi |
| habitat 6 | BR_G25/V. harveyi |
| habitat 6 | BR_G27/V. harveyi |
| habitat 6 | BR_G37/V. harveyi |
| habitat 6 | BR_G42/V. harveyi |
| habitat 6 | BR_G45/V. harveyi |
| habitat 6 | BR_G49/V. harveyi |
| habitat 6 | RB_74H/V. harveyi |
| habitat 6 | PD_A381/V. harveyi |
| habitat 6 | BR_G7/V. harveyi |
| habitat 6 | FP_2DA1/V. harveyi |
| habitat 6 | BR_G41/V. harveyi |
| habitat 6 | BE_33/V. harveyi |
| habitat 6 | BE_32/V. harveyi |
| habitat 6 | BR_G8/V. harveyi |
| habitat 6 | FP_1DA5/V. harveyi |
| habitat 6 | SA_R692/V. harveyi |
| habitat 6 | SH_R690/V. harveyi |
| habitat 6 | PD_A316/V. harveyi |
| habitat 6 | SA_R311/V. harveyi |
| habitat 6 | AW_P36G/V. harveyi |
| habitat 6 | PD_A308/V. harveyi |
| habitat 6 | BR_G22/V. harveyi |
| habitat 6 | AW_P36B/V. harveyi |
| habitat 6 | SA_R694/V. harveyi |
| habitat 6 | SA_R693/V. harveyi |
| habitat 6 | RB_74F/V. harveyi |
| habitat 6 | PS_A55/V. harveyi |
| habitat 6 | BR_G48/V. harveyi |
| habitat 6 | BR_G85/V. harveyi |
| habitat 6 | BR_G23/V. harveyi |
| habitat 6 | BR_G83/V. harveyi |
| habitat 6 | BR_G36/V. harveyi |
| habitat 6 | BR_G84/V. harveyi |
| habitat 6 | BR_G86/V. harveyi |
| habitat 6 | BR_G39/V. harveyi |
| habitat 6 | BR_G33/V. harveyi |
| habitat 6 | PD_A382/V. harveyi |
| habitat 6 | BR_G16/V. harveyi |
| habitat 6 | BR_G2/V. harveyi |
| habitat 6 | BE_28/V. harveyi |
| habitat 6 | BE_26/V. harveyi |
| habitat 6 | BE_25/V. harveyi |
| habitat 6 | BR_G40/V. harveyi |
| habitat 6 | AW_P36D/V. harveyi |
| habitat 6 | BR_G17/V. harveyi |
| habitat 6 | BR_G28/V. harveyi |
| habitat 6 | BR_G30/V. harveyi |
| habitat 6 | PD_A315/V. harveyi |
| habitat 6 | PD_A351/V. harveyi |
| habitat 6 | BR_G82/V. harveyi |
| habitat 6 | PD_A317/V. harveyi |
| habitat 6 | BR_G43/V. harveyi |
| habitat 6 | PD_A356/V. harveyi |
| habitat 6 | PD_A358/V. harveyi |
| habitat 6 | BR_G5/V. harveyi |
| habitat 6 | BR_G34/V. harveyi |
| habitat 6 | PS_A56/V. harveyi |
| habitat 6 | BR_G31/V. harveyi |
| habitat 6 | SH_R729/V. communis |
| habitat 6 | PS_A42/V. communis |
| habitat 6 | PS_A54/V. communis |
| habitat 6 | SH_R11/V. communis |
| habitat 6 | IB_PA2/V. communis |
| habitat 6 | BE_2/V. communis |
| habitat 6 | BR_G11/V. communis |
| habitat 6 | BR_G51/V. communis |
| habitat 6 | BR_G14/V. communis |
| habitat 6 | BR_G60/V. communis |
| habitat 6 | SH_R227/V. communis |
| habitat 6 | SH_R664/V. communis |
| habitat 6 | SH_R613/V. communis |
| habitat 6 | IB_PA3/V. communis |
| habitat 6 | AW_P26D/V. communis |
| habitat 6 | AW_P26E/V. communis |
| habitat 6 | AW_P26G/V. communis |
| habitat 6 | BR_G72/V. communis |
| habitat 6 | BR_G95/V. communis |
| habitat 6 | BR_G3/V. communis |
| habitat 6 | BE_22/V. communis |
| habitat 6 | PD_A301/V. communis |
| habitat 6 | BR_G1/V. communis |
| habitat 6 | BR_G54/V. communis |
| habitat 6 | BR_G50/V. communis |
| habitat 6 | BR_G70/V. communis |
| habitat 6 | PD_A313/V. communis |
| habitat 6 | BR_G52/V. communis |
| habitat 6 | BR_G61/V. communis |
| habitat 6 | SH_R680/V. communis |
| habitat 6 | AW_P4D/V. communis |
| habitat 6 | BR_G68/V. communis |
| habitat 6 | BR_G67/V. communis |
| habitat 6 | BR_G35/V. communis |
| habitat 6 | AW_P36A/V. communis |
| habitat 6 | PD_A372/V. communis |
| habitat 6 | PD_A376/V. communis |
| habitat 6 | BE_21/V. communis |
| habitat 6 | PD_A371/V. communis |
| habitat 6 | BR_G4/V. communis |
| habitat 6 | PS_A52/V. communis |
| habitat 6 | BR_G20/V. communis |
| habitat 6 | BE_23/V. communis |
| habitat 6 | BR_G53/V. communis |
| habitat 6 | PS_A44/V. communis |
| habitat 6 | PS_A37/V. communis |
| habitat 6 | PS_A51/V. communis |
| habitat 6 | PS_A46/V. communis |
| habitat 6 | PS_A41/V. communis |
| habitat 6 | PS_A53/V. communis |
| habitat 6 | PS_A38/V. communis |
| habitat 6 | PS_A40/V. communis |
| habitat 6 | PD_A332/V. communis |
| habitat 6 | PS_A39/V. communis |
| habitat 6 | PS_A45/V. communis |
| habitat 6 | PS_A43/V. communis |
| habitat 6 | PD_A331/V. communis |
| habitat 6 | PS_A47/V. communis |
| habitat 6 | BE_34/V. coralliilyticus |
| habitat 6 | BE_38/V. coralliilyticus |
| habitat 6 | BE_12/V. coralliilyticus |
| habitat 6 | BE_18/V. coralliilyticus |
| habitat 6 | BE_7/V. coralliilyticus |
| habitat 6 | FP_4SA1/V. sp |
| habitat 6 | PD_A15/V. tubiashii |
| habitat 6 | PD_A36/V. tubiashii |
| habitat 6 | BR_G91/V. tubiashii |
| habitat 6 | BR_G92/V. tubiashii |
| habitat 6 | BR_G55/V. tubiashii |
| habitat 6 | BE_11/V. agarivorans |
| habitat 6 | BR_G75/V. sp |
| habitat 6 | BR_G65/V. sp |
| habitat 6 | BR_G66/V. sp |
| habitat 6 | BR_G15/V. sp |
| habitat 6 | BE_37/V. pelagius |
| habitat 6 | BE_15/V. pelagius |
| habitat 6 | BR_G77/V. sp |
| habitat 6 | BR_G58/V. sp |
| habitat 6 | BR_G59/V. sp |
| habitat 6 | BR_G44/V. sp |
| habitat 6 | BR_G47/V. sp |
| habitat 6 | BE_35/V. sp |
| habitat 7 | SA_R78/V. variabilis |
| habitat 7 | SA_R240/V. sp |
| habitat 7 | SA_R12/V. meditarranei |
| habitat 7 | FP_1D2/V. xuii |
| habitat 7 | FP_2D2/V. nereis |
| habitat 7 | SH_R10/V. rotiferianus |
| habitat 7 | SH_R42/V. rotiferianus |
| habitat 7 | RB_74E/V. rotiferianus |
| habitat 7 | IB_PA1/V. rotiferianus |
| habitat 7 | SH_R682/V. rotiferianus |
| habitat 7 | FP_4S2/V. rotiferianus |
| habitat 7 | FP_4S1/V. rotiferianus |
| habitat 7 | FP_3S5/V. rotiferianus |
| habitat 7 | SA_R39/V. rotiferianus |
| habitat 7 | SA_R230/V. harveyi |
| habitat 7 | SA_R257/V. harveyi |
| habitat 7 | SA_R662/V. harveyi |
| habitat 7 | SA_R259/V. harveyi |
| habitat 7 | SA_R285/V. harveyi |
| habitat 7 | SA_R718/V. harveyi |
| habitat 7 | SA_R40/V. harveyi |
| habitat 7 | SH_R50/V. harveyi |
| habitat 7 | SH_R2/V. parahaemolyticus |
| habitat 7 | SA_R241/V. parahaemolyticus |
| habitat 7 | SA_R41/V. harveyi |
| habitat 7 | SA_R330/V. harveyi |
| habitat 7 | SA_R305/V. harveyi |
| habitat 7 | SA_R688/V. harveyi |
| habitat 7 | SA_R243/V. harveyi |
| habitat 7 | SA_R621/V. harveyi |
| habitat 7 | SA_R286/V. harveyi |
| habitat 7 | SA_R622/V. harveyi |
| habitat 7 | SA_R307/V. harveyi |
| habitat 7 | BR_G12/V. communis |
| habitat 7 | SA_R123/V. communis |
| habitat 7 | SH_R696/V. communis |
| habitat 7 | SH_R697/V. communis |
| habitat 7 | FP_4D3/V. communis |
| habitat 7 | BR_G78/V. communis |
| habitat 7 | SA_R260/V. communis |
| habitat 7 | SA_R86/V. communis |
| habitat 7 | FP_1D5/V. ponticus |
| habitat 7 | FP_3DA2/V. coralliilyticus |
| habitat 7 | FP_2DA3/V. coralliilyticus |
| habitat 7 | FP_3DA1/V. coralliilyticus |
| habitat 7 | FP_3DA3/V. coralliilyticus |
| habitat 7 | MB_45E/V. brasiliensis |
| habitat 7 | MB_45A/V. brasiliensis |
| habitat 7 | FP_4SA8/V.brasiliensis |
| habitat 7 | FP_4SA4/V. brasiliensis |
| habitat 7 | SA_R252/V. tubiashii |
| habitat 7 | AW_P68D/V. tubiashii |
| habitat 7 | SA_R35/V. tubiashii |
| habitat 7 | SA_R677/V. tubiashii |
| habitat 7 | SA_R676/V. tubiashii |
| habitat 7 | SA_R678/V. tubiashii |
| habitat 7 | IB_PA9/V. tubiashii |
| habitat 7 | SA_R229/V. tubiashii |
| habitat 7 | IB_PA4/V. tubiashii |
| habitat 7 | FP_1DA4/V. tubiashii |
| habitat 8 | PC_A205/V.shiloi |
| habitat 8 | SH_R720/V. meditarranei |
| habitat 8 | PC_A271/V.shiloi |
| habitat 8 | PC_A195/V.shiloi |
| habitat 8 | SH_R685/V. meditarranei |
| habitat 8 | PC_A94/V.shiloi |
| habitat 8 | PC_A194/V.shiloi |
| habitat 8 | PC_A169/V.shiloi |
| habitat 8 | SH_R686/V. meditarranei |
| habitat 8 | PC_A161/V.shiloi |
| habitat 8 | PC_A198/V.shiloi |
| habitat 8 | PC_A183/V.shiloi |
| habitat 8 | PC_A117/V.shiloi |
| habitat 8 | SH_R719/V. meditarranei |
| habitat 8 | SH_R660/V. meditarranei |
| habitat 8 | PC_A180/V.shiloi |
| habitat 8 | PC_A177/V.shiloi |
| habitat 8 | PC_A163/V.shiloi |
| habitat 8 | PC_A91/V.shiloi |
| habitat 8 | PC_A17/V.shiloi |
| habitat 8 | PC_A20/V.shiloi |
| habitat 8 | PC_A92/V.shiloi |
| habitat 8 | PC_A19/V.shiloi |
| habitat 8 | PC_A193/V.shiloi |
| habitat 8 | PC_A179/V.shiloi |
| habitat 8 | PC_A120/V.shiloi |
| habitat 8 | PC_A114/V.shiloi |
| habitat 8 | PC_A170/V.shiloi |
| habitat 8 | PC_A319/V.shiloi |
| habitat 8 | PC_A122/V.shiloi |
| habitat 8 | PC_A119/V.shiloi |
| habitat 8 | PC_A118/V.shiloi |
| habitat 8 | PC_A93/V.shiloi |
| habitat 8 | PC_A175/V.shiloi |
| habitat 8 | PC_A130/V.shiloi |
| habitat 8 | PC_A26/V.shiloi |
| habitat 8 | PC_A23/V.shiloi |
| habitat 8 | PC_A24/V.shiloi |
| habitat 8 | PC_A59/V.shiloi |
| habitat 8 | PC_A265/V.shiloi |
| habitat 8 | PC_A128/V.shiloi |
| habitat 8 | PC_A129/V.shiloi |
| habitat 8 | PC_A30/V.shiloi |
| habitat 8 | PC_A33/V.shiloi |
| habitat 8 | PC_A60/V.shiloi |
| habitat 8 | PC_A127/V.shiloi |
| habitat 8 | PC_A132/V.shiloi |
| habitat 8 | PC_A133/V.shiloi |
| habitat 8 | PC_A134/V.shiloi |
| habitat 8 | PC_A135/V.shiloi |
| habitat 8 | PC_A136/V.shiloi |
| habitat 8 | PC_A137/V.shiloi |
| habitat 8 | PC_A138/V.shiloi |
| habitat 8 | PC_A264/V.shiloi |
| habitat 8 | PC_A228/V.shiloi |
| habitat 8 | PC_A196/V.shiloi |
| habitat 8 | PC_A149/V.shiloi |
| habitat 8 | PC_A147/V.shiloi |
| habitat 8 | PC_A146/V.shiloi |
| habitat 8 | PC_A145/V.shiloi |
| habitat 8 | PC_A144/V.shiloi |
| habitat 8 | PC_A143/V.shiloi |
| habitat 8 | PC_A142/V.shiloi |
| habitat 8 | PC_A140/V.shiloi |
| habitat 8 | PC_A139/V.shiloi |
| habitat 8 | PC_A32/V.shiloi |
| habitat 8 | PC_A28/V.shiloi |
| habitat 8 | PC_A274/V.shiloi |
| habitat 8 | PC_A203/V.shiloi |
| habitat 8 | PC_A31/V.shiloi |
| habitat 8 | PC_A261/V.shiloi |
| habitat 8 | PC_A176/V.shiloi |
| habitat 8 | PC_A219/V.shiloi |
| habitat 8 | PC_A165/V.shiloi |
| habitat 8 | PC_A18/V.shiloi |
| habitat 8 | PC_A173/V.shiloi |
| habitat 8 | PC_A116/V.shiloi |
| habitat 8 | PC_A154/V.shiloi |
| habitat 8 | PC_A206/V.shiloi |
| habitat 8 | PC_A208/V.shiloi |
| habitat 8 | PC_A204/V.shiloi |
| habitat 8 | PC_A202/V.shiloi |
| habitat 8 | PC_A166/V.shiloi |
| habitat 8 | PC_A150/V.shiloi |
| habitat 8 | PC_A159/V.shiloi |
| habitat 8 | PC_A164/V.shiloi |
| habitat 8 | PC_A215/V.shiloi |
| habitat 8 | PD_A294/V.shiloi |
| habitat 8 | PC_A230/V.shiloi |
| habitat 8 | PC_A229/V.shiloi |
| habitat 8 | PC_A226/V.shiloi |
| habitat 8 | PC_A211/V.shiloi |
| habitat 8 | PC_A210/V.shiloi |
| habitat 8 | PC_A199/V.shiloi |
| habitat 8 | PC_A123/V.shiloi |
| habitat 8 | PC_A185/V.shiloi |
| habitat 8 | PC_A200/V.shiloi |
| habitat 8 | PC_A207/V.shiloi |
| habitat 8 | PC_A201/V.shiloi |
| habitat 8 | PC_A212/V.shiloi |
| habitat 8 | PC_A184/V.shiloi |
| habitat 8 | PC_A131/V.shiloi |
| habitat 8 | PC_A158/V.shiloi |
| habitat 8 | PC_A266/V.shiloi |
| habitat 8 | PC_A99/V.shiloi |
| habitat 8 | PC_A224/V.shiloi |
| habitat 8 | SH_R661/V. meditarranei |
| habitat 8 | PC_A263/V.shiloi |
| habitat 8 | PC_A112/V.shiloi |
| habitat 8 | PC_A148/V.shiloi |
| habitat 8 | PC_A182/V.shiloi |
| habitat 8 | PC_A171/V.shiloi |
| habitat 8 | PC_A218/V.shiloi |
| habitat 8 | PC_A160/V.shiloi |
| habitat 8 | PC_A178/V.shiloi |
| habitat 8 | PC_A162/V.shiloi |
| habitat 8 | PC_A214/V.shiloi |
| habitat 8 | PC_A174/V.shiloi |
| habitat 8 | PC_A172/V.shiloi |
| habitat 8 | PC_A167/V.shiloi |
| habitat 8 | PC_A113/V.shiloi |
| habitat 8 | PC_A209/V.shiloi |
| habitat 8 | PC_A197/V.shiloi |
| habitat 8 | PC_A217/V.shiloi |
| habitat 8 | PC_A151/V.shiloi |
| habitat 8 | PC_A152/V.shiloi |
| habitat 8 | PC_A227/V.shiloi |
| habitat 8 | PC_A168/V.shiloi |
| habitat 8 | PC_A153/V.shiloi |
| habitat 8 | PC_A220/V.shiloi |
| habitat 8 | PC_A181/V.shiloi |
| habitat 8 | PC_A221/V.shiloi |
| habitat 8 | PC_A157/V.shiloi |
| habitat 8 | SH_R713/V. meditarranei |
| habitat 8 | PC_A121/V.shiloi |
| habitat 8 | PC_A125/V.shiloi |
| habitat 8 | PC_A124/V.shiloi |
| habitat 8 | PC_A126/V.shiloi |
| habitat 8 | PC_A16/V.shiloi |
| habitat 8 | PC_A155/V.shiloi |
| habitat 8 | PC_A216/V.shiloi |
| habitat 8 | PC_A192/V.shiloi |
| habitat 8 | SH_R638/V. meditarranei |
| habitat 8 | SH_R639/V. meditarranei |
| habitat 8 | PC_A213/V. furnissii |
| habitat 8 | PC_A225/V. furnissii |
| habitat 8 | PC_A223/V. furnissii |
| habitat 8 | PC_A222/V. furnissii |
